# The effect of clinically relevant changes in extracellular electrolyte concentrations on human atrial arrhythmias

**DOI:** 10.1038/s43856-025-01260-4

**Published:** 2025-12-02

**Authors:** Cesare Corrado, Caroline H. Roney, Sanjiv M. Narayan, Wayne R. Giles, Steven A. Niederer

**Affiliations:** 1 National Heart and Lung Institute, Imperial College London, London, UK; 2 School of Engineering and Materials Science, Queen Mary University of London, London, UK; 3 Department of Medicine and Cardiovascular Institute, Stanford University, Palo Alto, CA USA; 4 Cumming School of Medicine, Department of Physiology & Pharmacology, University of Calgary, Calgary, Canada

**Keywords:** Interventional cardiology, Atrial fibrillation, Medical research

## Abstract

**Background:**

Patients with recent-onset atrial fibrillation (AF) frequently present with plasma electrolyte imbalances. Low plasma concentrations of potassium (hypokalaemia), and more recently low plasma concentrations of sodium (hyponatremia), have both been shown to contribute to a pro-arrhythmic substrate and may affect the success of restoring the normal rhythm (cardioversion). However, the mechanistic effects of these electrolyte alterations on atrial electrophysiology remain incompletely understood. This study aims to investigate how clinically relevant variations in extracellular electrolyte concentrations influence human atrial electrical activity and arrhythmia initiation.

**Methods:**

We applied our cardiac digital twin methodology to a cohort of 100 atrial fibrillation patients (43 paroxysmal, 41 persistent, 16 long-standing persistent). For each patient-specific model, we simulated sinus rhythm and AF induction under baseline and 30 distinct combinations of extracellular potassium, sodium and calcium concentrations. Global sensitivity analysis and machine learning were used to quantify how these electrolyte alterations affect key electrophysiological markers, including action potential duration, resting membrane potential (RMP), and conduction velocity (CV), and the induction and maintenance of AF.

**Results:**

Here we show that our computational framework accurately replicates the experimentally observed sensitivity of human atrial electrophysiology to electrolyte variations. Hyponatremia significantly modifies the action potential waveform, thereby promoting AF sustainability, while hypokalaemia predominantly alters the RMP and thus CV, and only moderately increases AF inducibility.

**Conclusions:**

The combination of clinical data sets and multiscale computational analyses yields insights into cellular and tissue-level mechanisms for AF as well as suggesting personalised approaches for management and treatment.

## Plain language summary

Atrial fibrillation (AF) is the most common irregular heartbeat in adults. Many studies look at what triggers AF, often in patients after surgery or on dialysis. Our study focused on small changes in blood salts—potassium, sodium, and calcium—that often happen in everyday medical situations. Using information from 100 human hearts, we tested how these changes affect normal heart activity and the chance of AF starting. We used computer models and machine learning to study the results. We found that even small changes in sodium and potassium can disturb the heart’s electrical signals, slow how signals spread, and make AF more likely. These findings could help doctors better understand and prevent AF linked to common medical conditions.

## Abstract (web summary)

Corrado et al. use cardiac digital twins from 100 atrial fibrillation patients to evaluate how electrolyte imbalances influence atrial electrophysiology and arrhythmia risk. Hyponatremia is identified as a key determinant of sustained AF, while hypokalemia modestly increases the likelihood of arrhythmia onset.

## Introduction

Atrial fibrillation (AF) is a supraventricular arrhythmia marked by disorganised atrial activation^1^, and impaired contractile function of the heart^2^. Maintained or chronic AF can increase the risk of stroke^3,4^. Cardioversion procedures are often recommended for treatment of recent-onset AF^5^. Approximately 20% of these patients, when admitted, have serum potassium levels, [K^+^]_o_ somewhat lower than normal, defined as hypokalaemia^6^. In addition, approximately 4% of them present [K^+^]_o_ levels that are higher than normal, or hyperkalaemia^7^. Both conditions are known to promote arrhythmias^8,9^ and may increase mortality risk^10^. It is also known that patients being treated for heart failure^11^ or chronic kidney disease^12^ are prone to having low plasma sodium levels, [Na^+^]_o_. This hyponatremia also may increase the risk of developing AF^13,14^; in part through its effects on the action potential waveform^15^. When maintained, abnormal [Na^+^]_o_ levels are associated with increased mortality^16^. Clinical studies have shown that correcting either or both of these plasma electrolyte abnormalities can convert either AF or atrial flutter (AFL) to sinus rhythm^17^. In addition, there is evidence that restoring [K^+^]_o_ to normal levels can increase the effectiveness of cardioversion procedures^18^.

Our group, and others, have successfully obtained and analysed digitised cohorts of human atrial data, with the initial goals of further understanding the underlying mechanisms for atrial rhythm disturbances and attempting to predict their occurrence/reoccurrence. Previous, detailed and multiscale computational work published by the Severi and Dossel groups^19^ has addressed key aspects of the electrophysiological effects of changes in plasma electrolytes on the human atrial action potential, its conduction velocity, and the ability of this in silico bi-atrial preparation to generate altered P-waves. These findings constitute an important and informative background for our study. Moreover, the emerging ‘digital twin’ paradigm has advanced the present understanding of atrial arrhythmia mechanisms^20–24^, and has also contributed to earlier and more reliable identification of this proarrhythmic substrate for AF^25^. In combination, these advances have already led to the optimisation of clinical interventions, including radiofrequency ablation^26^.

In this study, our primary goal was to determine whether relatively small ( ± 25%) changes in plasma electrolytes can alter human atrial arrhythmia burden. Our studies have been done under control or baseline conditions in healthy atria; and when the left atrial substrate has been altered by two known proarrhythmic factors: i) progressive fibrosis and/or ii) atrial enlargement.

Most previous computational studies concerning electrolyte imbalances have relied on either single myocyte models (0-D) or one-dimensional (1-D) atrial tissue strands^27–30^. In contrast, our analysis of the effects of clinically relevant electrolyte changes uses a multi-scale approach: i) the single isolated atrial myocyte (0-D), ii) a strand or trabeculum of human atrial tissue (1-D), and iii) 100 patient-specific left atrial samples. In each of these targeted settings, patterns of results from healthy atria preparations with normal plasma electrolyte levels have been compared and contrasted with data sets obtained after altering one of the three plasma electrolyte (Na^+^, K^+^, and/or Ca^2+^) levels. Finally, as mentioned, these conditions and protocols are repeated in the settings of simulated atrial fibrosis or enlargement.

To accomplish this, we first needed to modify the original version of the Courtemanche^31^ human atrial action potential model. Specifically, the mathematical expressions for the potassium currents IKr (rapid delayed rectifier) and IK1 (inward rectifier) were modified so that the model could accurately reproduce the experimentally observed changes in the resting membrane potential (RMP) and the action potential duration (APD) in response to even very small variations in [K^+^]_o_ levels. Our analysis was based on assessing the global sensitivity of defined outputs (for example, changes in APD or conduction velocity, CV) to selected changes in plasma electrolytes within the range +/−25%. This was complemented by the use of machine learning procedures to detect and classify the induced AF in each of the 100 human atrial data sets. In a previous paper^25^, we validated a cohort of *n* = 100 personalised left atrial digital twins^25^ and used this data set to attempt to predict the long-term recurrence of atrial fibrillation following an initial ablation procedure. Although this cohort took into account key aspects of atrial substrate inter-individual variability, the plasma electrolyte concentrations were all fixed at baseline levels. In the present study, we have used this same cohort. The effects of clinically relevant alterations ( ± 25%) in [K^+^]_o_, [Na^+^]_o_, and [Ca^2+^]_o_ on the action potential (AP) waveforms, the RMP, CV in simulated atrial tissue strips or trabeculate, and their pro-arrhythmic effects on the entire atria were investigated. In addition, the effects of these changes, alone and in combination, were evaluated in the settings of simulated atrial fibrosis and/or after considering the differences in the surface area of the atria exhibited by the clinical cohort.

Our simulations and analyses reveal that variations in extracellular sodium concentration ([Na^+^]_o_) significantly affect the sustainability of atrial arrhythmias, primarily through pronounced changes (collapse) in the action potential plateau. In addition, and consistent with prior studies, even minor alterations in extracellular potassium concentration ([K^+^]_o_) alter the conduction velocity by shifting the resting membrane potential, thereby increasing arrhythmia inducibility, especially in the setting of augmented fibrosis. In contrast, the modest variations in extracellular calcium concentration ([Ca^2+^]_o_) that are examined in this study have no notable effects on atrial arrhythmia inducibility or its duration.

## Methods

**Fig. 1:**
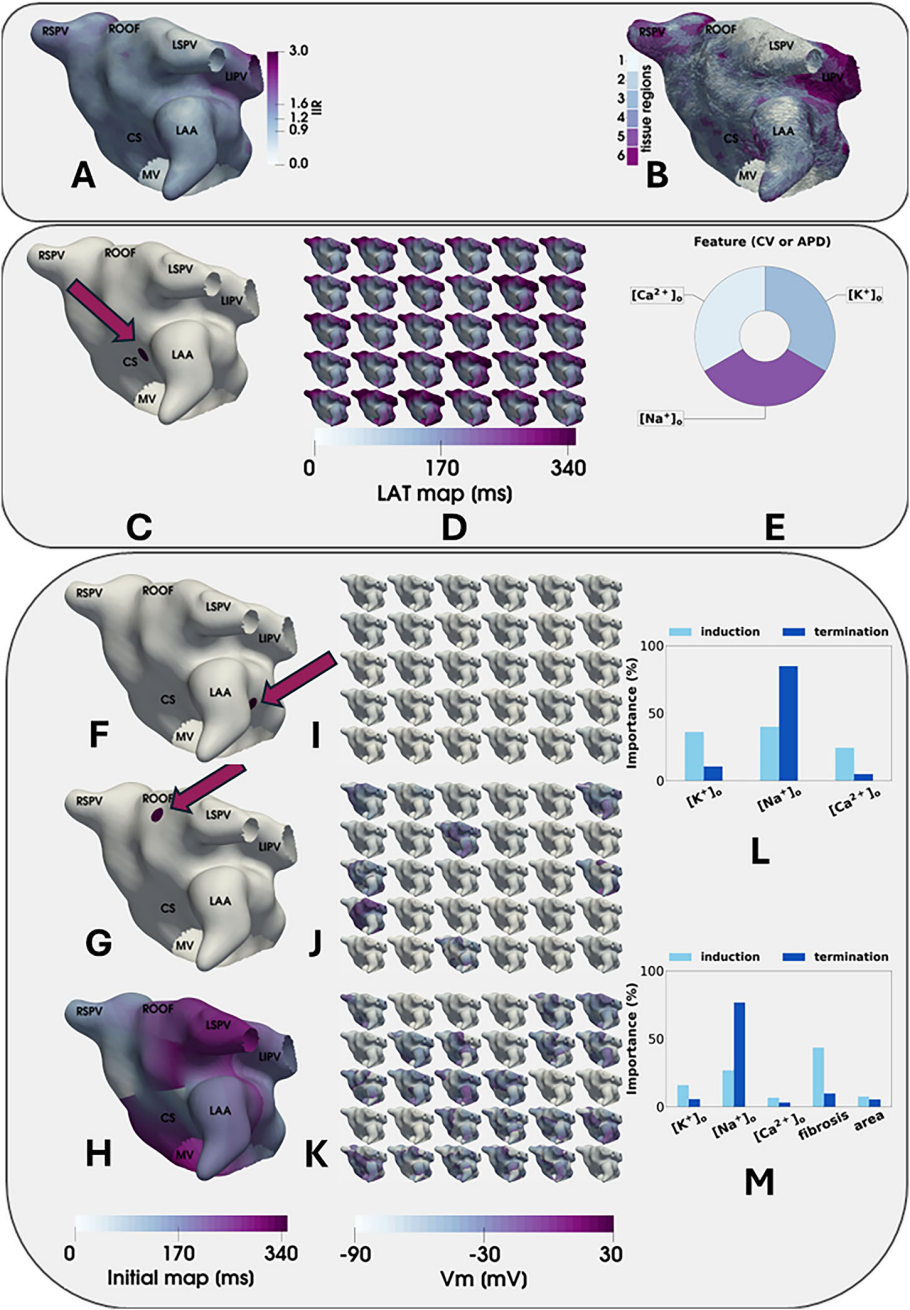
Workflow diagram illustrating the rationale and the main components of this study. MRI data sets were used to calculate intensity ratios (IIR) for each human atrial tissue (**A**). These patterns of results were used to define six different tissue types within each such heterogeneous atrial substrate (**B**). Paced activation was achieved by delivering a stimulus in the region localised by the arrow in (**C**) that is located near the coronary sinus (CS) in each of these 100 atrial samples. This pacing manoeuvre was then repeated for each of 30 selected individual plasma levels of [Na^+^]_o_, [K^+^]_o_, or [Ca^+2^]_o_. From each of these paced activation data sets, a corresponding local activation time (LAT) map was computed (**D**). Estimates of the whole organ (atrial) sensitivity of both conduction velocity (CV) and action potential duration (APD) to each selected plasma electrolyte level were then obtained (**E**). These findings were transformed into output features by calculating median CV and APD values and their inter-percentile ranges for each human atrial tissue sample. Using these same sets of electrolyte concentrations for each personalised atrial sample, we then assessed the ability to induce and maintain atrial arrhythmias for a time window lasting 5 s after induction. Arrhythmia induction was accomplished by using three different protocols: i) burst pacing in the proximity of the left atrial appendage (LAA) localised by the arrow in panel **F**(**I**), burst pacing on the atrial roof (AR) localised by the arrow in panel **G** (**J**), and iii) an imposed initial state consisting of four spirals waves each having a different chirality (**H, K**). These simulated results were then used to determine whether: (i) burst pacing on the atrial roof, (ii) burst pacing near the LAA, or (iii) burst pacing on any of these two locations induced arrhythmias, and if these arrhythmias lasted more than 5 seconds. Thereafter, we determined whether each of the selected electrolytes concentration levels could alter arrhythmia induction or maintenance for each atrial digital twin (**L**). Finally, this procedure was repeated over the entire data set after augmenting the input features with the amount of fibrosis and the left atrial surface area (**M**).

The workflow scheme that guided this study is presented in Fig. 1. As illustrated in Fig. 1A, B, the human atrial tissue for each subject (*n* = 100) has been characterised using the late gadolinium enhancement (LGE) magnetic resonance images (MRI), with each expressed as a segmented image intensity ratios (IIR). The arrow in Fig. 1C, localises the paced activation protocols initiated by applying stimuli in the proximity of the coronary sinus (CS) of each human atrial preparation. This dataset was used as a baseline for comparison with atrial arrhythmia (AF) responses. Selected changes in extracellular or plasma electrolyte concentrations (see Fig. 1E) were assessed, one at a time, in terms of their ability to alter the action potential duration (APD) and/or the conduction velocity (CV) utilising the simulated responses in each atrial tissue (Fig. 1D). A variance-based semiquantitative sensitivity analysis method, described in detail in supplement (section ‘Variance-based global sensitivity analysis’), was utilised to express these patterns of results.

In each atrial preparation, arrhythmias were induced by applying burst pacing in the proximity of the left atrial appendage (Fig. 1F), the atrial roof (Fig. 1G), or by providing initial conditions consisting of four spiral voltage waves (Fig. 1H). In this study, each of these three AF initiation protocols was applied to each human atrial tissue sample (*n* = 100) after a fixed series of individual changes in plasma electrolyte concentrations had been selected and applied.

Using the data sets generated by each of these simulations, information was extracted so that each induced arrhythmia could be grouped into four classifications. These were arrhythmias induced by burst pacing i) near the left atrial appendage, ii) on the atrial roof, or iii) by burst pacing at either of these two locations. In addition, prolonged arrhythmias that terminated within five seconds after initiation were separately tabulated and denoted as class iv).

We next utilised machine learning methods by training one classifier for each of these four categories (or phenotypes) of rhythm disturbance that was induced in each human atrial preparation (*n* = 100). Our main goal using this approach was to be able to estimate whether each selected plasma electrolyte concentration could modulate the predicted outcomes (Fig. 1I–K), as judged by arrhythmia induction and sustainability as termination criterion (Fig. 1L, M).

The final part of this study consisted of analogous studies done under conditions of i) simulated atrial fibrosis and ii) atrial enlargement. For this initial investigation (recognising the significant computational requirements), we trained only one classifier (for example, termination) using all 100 human atrial samples. We then compared this pattern of results to the patterns obtained under conditions wherein the input features have been altered to replicate some aspects of the effects (or burden) of atrial fibrosis or altered atrial surface area (Fig. 1M).

### Human atrial myocyte action potential model

The original Courtemanche (CRN) human atrial action potential model^31^ was modified. As we have demonstrated previously, this was done to ensure that the output(s) can accurately simulate the known nonlinear effects of even small changes in the extracellular potassium concentration, [K^+^]_o,_ on both the rapid delayed rectifier potassium current, IKr, and the inwardly rectifying background potassium current, IK1^32^. These modifications allow the CRN + + model to reproduce the changes in both the RMP and the APD (specifically late repolarisation), generated by alterations in [K^+^]_o_ (see the supplement section 'The modified Courtemanche (CRN) model of the human atrial action potential'). Throughout this study, the CRN + + model applied to each human atrial tissue was run in the monodomain configuration.

### Patient-specific human atrial datasets

For this study, we made use of our previously defined cohort of 100 atrial digital twins, each derived from LGE-MRI images^25^. We used data from a public repository based on patient data collected under ethical approval granted by the regional ethics committee (17/LO/0150 and 15/LO/1803, granted by the London - Westminster Research Ethics Committee), and subjects gave informed consent. Each of these meshes was refined to achieve a maximum edge length of 340 microns. As we have done in our previous paper, the approach for modelling inherent atrial tissue heterogeneity was based on patient and tissue region-specific LGE MRI image intensity ratios (IIR). Specifically, IIR values were used to subdivide each atrial tissue into six different regions or subtypes (Fig. 1A). Table S-1 in the supplement summarises the related longitudinal (σ_il_) and transverse (σ_it_) tissue conductivities. Fibrotic regions in each human atrial tissue sample were defined as having IIR values greater than 1.22. These individual fibrosis regions/indices ranged between 0% and 70% of the total atrial tissue, with the mean being 21% ± 15% (std). As shown in Table S-1, based on our previous studies, fibrotic atria tissue electrophysiological properties were modelled by decreasing the following maximal conductances^33,34^ for the sodium current, INa, (GNa by 40%), the inwardly rectifying background K^+^ current, IK1, (GK1 by 50%), and the L-type calcium current, ICaL, (GCaL by 50%). Note that using this approach, individual fibroblast properties can’t be accounted for. Thus, their electrotonic depolarising influence on the atrial myocyte syncytium was not considered^35^.

### Extracellular electrolyte concentrations

The main goal of this study was to rigorously assess the effects of small changes in extracellular or plasma electrolyte concentrations on human atrial electrophysiological properties, including arrhythmia induction and sustainability. Our starting or control conditions therefore employed ranges and reference values for electrolyte that have been reported in human plasma^36–40^. These are [K^+^]_o_ = 4.5 ± 1.5 (33%) mM; [Na^+^]_o_ = 140 ± 35 (25%) mM; [Ca^2+^]_o_ = 1.8 ± 0.45 (25%) mM. The CRN + + model of the human atrial action potential adequately reconstructed the known action potential waveform changes resulting from these electrolyte levels (see the supplement section ‘The modified Courtemanche (CRN) model of the human atrial action potential’ and Supplementary. Fig. S-1). Latin hypercube sampling (LHS) was used to obtain 30 combinations of extracellular electrolyte concentrations that were utilized and compared with control or reference values in this study (see Supplementary. Table S2).

### Numerical simulations and analyses of atrial response to paced activation protocols

In Part I of this study, the effects of each LHS-derived electrolyte level or combinations (see Supplementary Table S2) were evaluated in response to coronary sinus (CS) pacing (to mimic clinical procedures), by comparing APD and CV values with respect to data obtained in the presence of reference or baseline plasma electrolyte concentrations. We identified the CS using the universal atrial coordinates (UAC)^23^. It was identified as the region within 0.5 ≤ UAC1 ≤ 0.7; 0.8 ≤ UAC2 ≤ 0.9. This reference system was also used to identify each of the other locations for applying stimuli to our atrial twin samples.

For each of these protocols, we initialised the modified Courtemanche atrial myocyte model (CRN + +) using the pre-pace functionality provided by the software package, openCARP^41^. Specifically, a stimulus train lasting 15 min^28^ was delivered at a 1000 ms cycle length (CL) to each atrial tissue sample, thus approximating a limit cycle.

Atrial activation was then simulated in all cases by pacing the CS region with two stimuli 1000 ms apart. Values for local activation times (LAT) were calculated using the activation waveform due to the second stimulus. We obtained each LAT value by using the time derivative of the conducted transmembrane potential; specifically, by calculating the time point when the rate of change reached its maximum (${LAT}={{\rm{argmax}}}\left(\tfrac{\partial {Vm}}{\partial t}\right)$). The corresponding local repolarisation time (LRT) was measured as the time point when the repolarising transmembrane potential 'recovered' by 90%. Using the gradient of the LAT map for each atrial sample, we constructed a patient-specific map of the magnitude of local CV. Second, using the difference between LRT and LAT values, we computed a corresponding map of APD values(in fact, the APD_90_). From these data sets, we extracted the patient-specific median values (mAPD, mCV) of interest, and then calculated their variability as inter-percentile interval values (between 97.5 and 2.5) percentiles, denoted as DAPD and DCV, respectively.

Finally, for each selected electrolyte concentration, we computed the relative variation of (mAPD, mCV, DAPD, and DCV) relative to those obtained using the reference or baseline electrolyte concentrations. These relative variation values were used as the quantities of interest (QoI) and their sensitivity to each selected [Ca^2+^]_o_, [K^+^]_o_, and [Na^+^]_o_ level was obtained using the global sensitivity analysis (GSA) approach that is described in the supplement (section ‘Variance-based global sensitivity analysis’)

### Numerical simulations and analyses of human atrial arrhythmia induction and longevity

For each human atrial digital twin (*n* = 100), a total of 31 models were created and analysed. Of these, 30 were generated by the LHS procedure, and 1 (the control or baseline set) was based on the reference extracellular electrolyte values. In these sets of simulations, patient-specific atria arrhythmias were induced using three different protocols. In the first two protocols, the following procedure was adopted^42^. The Courtemanche human atrial model was initialised in each of the six designated atrial regions by pacing it for 15 minutes at CL of 700 ms, to reach a limit cycle^28^. The arrhythmia induction protocol was applied immediately. It consisted of 5 consecutive stimuli applied in the proximity of the coronary simus, at CL of 700 ms followed by 400 ms delay, and finally a short burst-pacing challenge consisting of a train of 5 stimuli delivered at a CL of 160 ms in an ectopic location. This arrhythmia induction procedure was applied at 2 different locations (protocols 1 and 2) that were selected using the UAC system for each of the 100 atrial samples. These locations were immediately below the left atrial appendage (LAA), at 0.9 ≤ UAC1 ≤ 0.91 and 0.9 ≤ UAC2 ≤ 0.91 (Fig. 1F), and on the atrial roof, at 0.48 ≤ UAC1 ≤ 0.49 and 0.48 ≤ UAC2 ≤ 0.49 (Fig. 1G). In contrast, in the third protocol, each atrial tissue was first paced for 15 min at CL of 300 ms, to reach a limit cycle^28^; and then an ‘induction initial condition’ was immediately applied (Fig. 1H). This consisted of 4 spiral voltage waves^43^, computed as we have described in detail previously^23,26^. Each induced arrhythmia was tracked/recorded for a total of 5 seconds to allow descriptive parameters that are consistent with analogous data sets published by other groups to be obtained^21,22,33,34^.

Making use of the measurements and analytical methods described in the supplement (section ‘Criteria used to identify induced human atrial arrhythmias’), for each of the 31 human atrial models, we determined if it was possible to induce an arrhythmia in response to protocols 1 or 2, or either of them. We also catalogued whether the induced arrhythmias terminated within 5 seconds after their initiation.

In part III of this study, machine learning software was employed. We first trained classifiers (logit regression, random forest, and gradient boost) as described in the supplement (section ‘Induced human atrial arrhythmia classifiers’). The random forest classifier was selected to generate all the results that we report, since it achieved the best performances. For our analysis, two classifiers were trained. One classifier was trained for each digital twin, using the pattern of responses to changes in the 3 electrolyte concentrations as input parameters. The second classifier was trained over the entire cohort, after augmenting the input parameters with 2 additional features. The first feature attempted to account for the variable fibrosis burden (fibrotic surface/total surface); and the second feature captured the differences in the surface areas of these atrial samples. As illustrated in Fig. 7, Shapley analysis and permutation feature importance were used to determine which extracellular electrolyte concentrations had the largest proarrhythmic impact, measured in terms of increased ability to initiate or sustain the patient-specific atria arrhythmia. All Shapley indices were computed using the library Shap^44^ and the results are presented using a violin plot format. We note that for each patient-specific atrial dataset, this analysis was performed only when each binary classifier included at least two responses.

## Results

### Global sensitivity analysis (GSA) of electrolyte-induced changes in the APD and RMP of an isolated human atrial myocyte

**Fig. 2:**
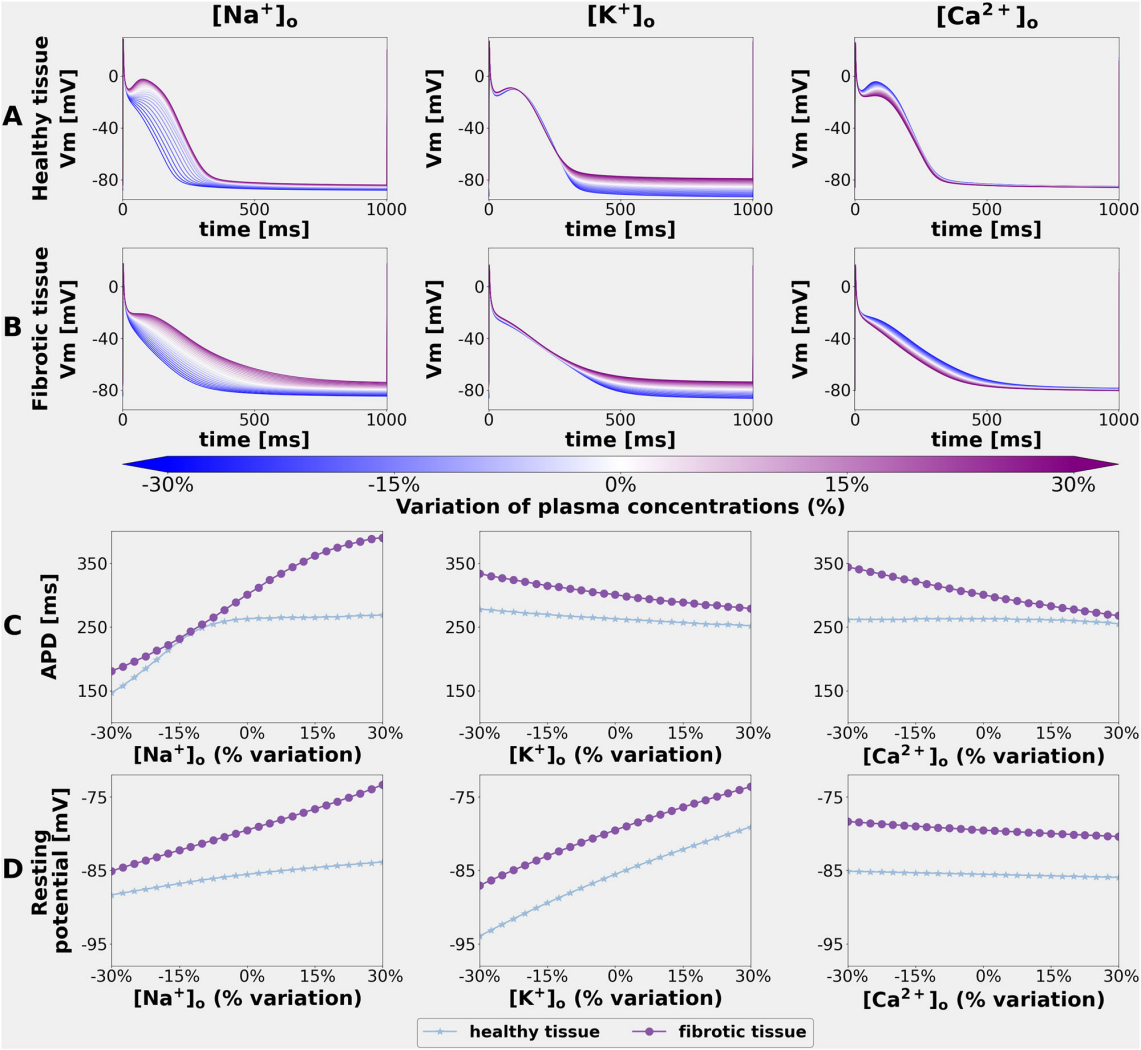
Effects of selected alterations in plasma electrolytes on the human atrial myocyte RMP, APD, and action potential waveforms at baseline (healthy tissue) and in the setting of atrial fibrosis. The superimposed action potentials in Row **A** (left column) show the effects of ± 25% alterations in [Na^+^]_o_ on the action potential waveform and RMP using the colour coding shown at the bottom as a guide for the specifics of these changes. Row **B** (left column) shows analogous data obtained after altering the model to simulate atrial fibrosis. Note that decreasing [Na^+^]_o_ markedly shortens the action potential under both conditions. Note also that 25% increases in [Na^+^]_o_ have a negligible effect under baseline conditions (Panel 1 **A** left) but significantly depolarise RMP in atrial fibrosis. Data sets obtained after ± 25% changes in [K^+^]_o_ are shown in the middle columns of Rows (**A**, **B**). Under control or baseline conditions, the selected increases in [K^+^]_o_ result in a modest depolarisation of RMP and small alterations in APD (**C**). In contrast, decreases in [K^+^]_o_ produce a marked hyperpolarisation of RMP both at baseline and in the setting of fibrosis. The two sets of colour-coded data in Rows (**C**, **D**) provide numerical summaries of the effects of these changes in [Na^+^]_o_ and [K^+^]_o_. As shown in the right-hand column, [Ca^2+^]_o_ was also altered by ± 25%; however, these manoeuvres produced only relatively small changes ( ± 5–7 mV) at the peak height of the AP plateau under baseline conditions.

**Fig. 3:**
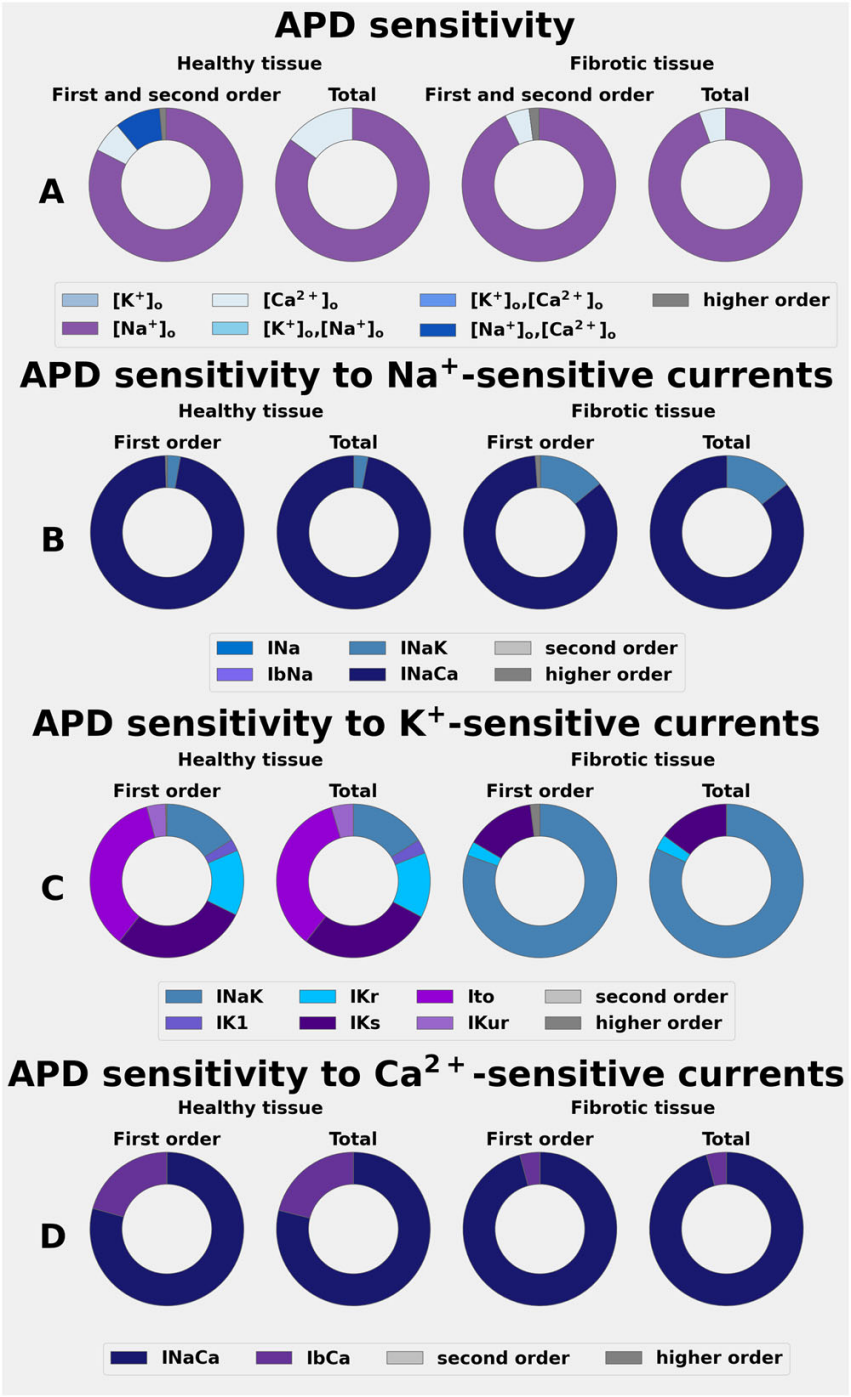
Demonstration of the sensitivity of APD changes effects after alterations in plasma electrolytes under baseline conditions and in the setting of fibrosis. The results, based on global sensitivity analysis and expressed in terms first order and total effects are shown in Rows (**A**–**D**). Individual outputs colour-coded to denote the specific ion channel or carrier-mediated transmembrane ionic current of interest. In each Row of this Figure, data obtained when studying the control or baseline model is shown in the two columns on the left, while analogous data sets obtained from fibrotic tissue are shown in the two columns on the right. In (**A**), effects in healthy tissue (two left columns) clearly show that the observed changes in APD result mainly from selected alterations in [Na^+^]_o_, and also emphasise that the combined changes [Na^+^]_o_,[Ca^2+^]_o_ make a significant contribution. The data sets in Row **C** summarise the relative importance of [K^+^]_o_-sensitive currents on the (minor) alterations produced on the APD. Note, however, that in the setting of fibrosis (two right-hand Columns), the electrogenic outward current generated by the Na^+^/K^+^ pump, INaK, also makes a small contribution to the changes in APD. Row **D** shows that although the changes in APD are small in response to alterations in [Ca^2+^]_o_, two currents, IbCa and the electrogenic Na^+^/Ca^2+^ exchanger, can result in small APD changes.

In Part I of this study, the effects of ±25% changes in [Na^+^]_o_, [K^+^]_o,_ and [Ca^2+^]_o_ on human APD and RMP were assessed using global sensitivity analyses (GSA). Each electrolyte level was varied independently, and the effects were studied both in healthy atrial myocytes (Fig. 2, Row A) and after simulating a spatially heterogeneous fibrosis (Fig. 2, Row B). Changes in atrial APD (APD_70_) sensitivity to each electrolyte change were then expressed in terms of Sobol indices (Fig. 3). The selected Sobol indices measure the primary effect (first order) of each individual electrolyte change (e.g., how the output changes when only that species varies), and also the secondary or second order effects due to the interactions between selected electrolytes single levels (e.g., how the output changes when two electrolytes vary simultaneously). First and higher-order effects describe the output variations normalised by the output variations (variance); hence, they sum up to 1. The Sobol indices denoted ‘total’ are generated from the combined effects of each combination of 3 plasma electrolyte levels and express, for each electrolyte, the total variance produced in the output when the interactions with the other species are also taken into account. Sobol indices are shown using doughnut plots, representing the fractional contribution of each input in producing the observed perturbation in the output (e.g., an input that covers half of the doughnut produces 50% of the observed variation).

As expected, even very small alterations in [K^+^]_o_ significantly changed the RMP of the human atrial myocyte^45–49^. These results are illustrated in Fig. 2, (middle column row A). Analogous results obtained in response to these same alterations in [K^+^]_o_ in the settings of simulated atrial fibrosis are illustrated in Fig. 2, Row D. Note that in either healthy or fibrotic myocytes, these changes in [K^+^]_o_ produced no significant APD changes under baseline conditions (Fig. 2, row A), while tending to lengthen APD_70_ in the setting of fibrosis. In contrast, ± 25% [Na^+^]_o_ variations resulted in substantial AP waveform changes (suppression of the AP plateau) and quite marked shortening of the APD under both baseline and fibrotic conditions (Fig. 2, left column, rows A, B and C). In addition, increasing [Na^+^]_o_ resulted in a small depolarization of the RMP at baseline, and this effect was enhanced in the setting of fibrosis (Fig. 2, left column, rows B and D). In contrast, the selected changes in [Ca^2+^]_o_ had such small effects on RMP and APD (Fig. 2 right column), that they were judged to be insignificant.

Additionally, analyses of the changes shown in Fig. 2, expressed in terms of global sensitivity parameters (Sobol indices), are presented in Fig. 3. As shown in Panel A, GSA identified the selected plasma levels of [Na^+^]_o_ as the primary driver for alterations in the AP waveform, measured as APD changes. Moreover, when these effects were studied and expressed in terms of sensitivity to the transmembrane currents in the CRN + + model that are dominated by Na^+^ fluxes or permeability, the Sobol indices identify the Na^+^/Ca^2+^ exchanger current (INaCa) as being the most important causative variable. However, inspection of Fig. 3, Rows A and B, also shows that changes in [Na^+^]_o_ can influence APD, apparently primarily through alterations in the outward electrogenic current produced by the Na^+^/K^+^ pump (INaK). In summary, the marked shortening of the action potential and the collapse of its plateau arise mainly from increased INaCa and INaK. Both of these effects are augmented in the setting of atrial fibrosis as illustrated in the two right-hand columns of Fig. 3, Panels A and B (see Discussion). The data in Row D of Fig. 3 illustrate the effects of changes in [Ca^2+^]_o_ expressed in terms of transmembrane currents carried or mediated primarily by the permeant divalent Ca^2+^. As noted, the effects on APD and/or RMP were very small, likely to be functionally insignificant, and hence no further mechanistic insights can be presented.

Although the focus of this study was on electrolyte-induced changes in human atrial electrophysiology, we also did similar investigations of these same changes in the healthy human ventricle. These data sets are shown in the supplement (Figs. S-3 and S-4) and are provided only for the purposes of an initial comparison and contrast.

### Global sensitivity analysis of electrolyte-induced changes in the conduction velocity in human atrial tissue

**Fig. 4:**
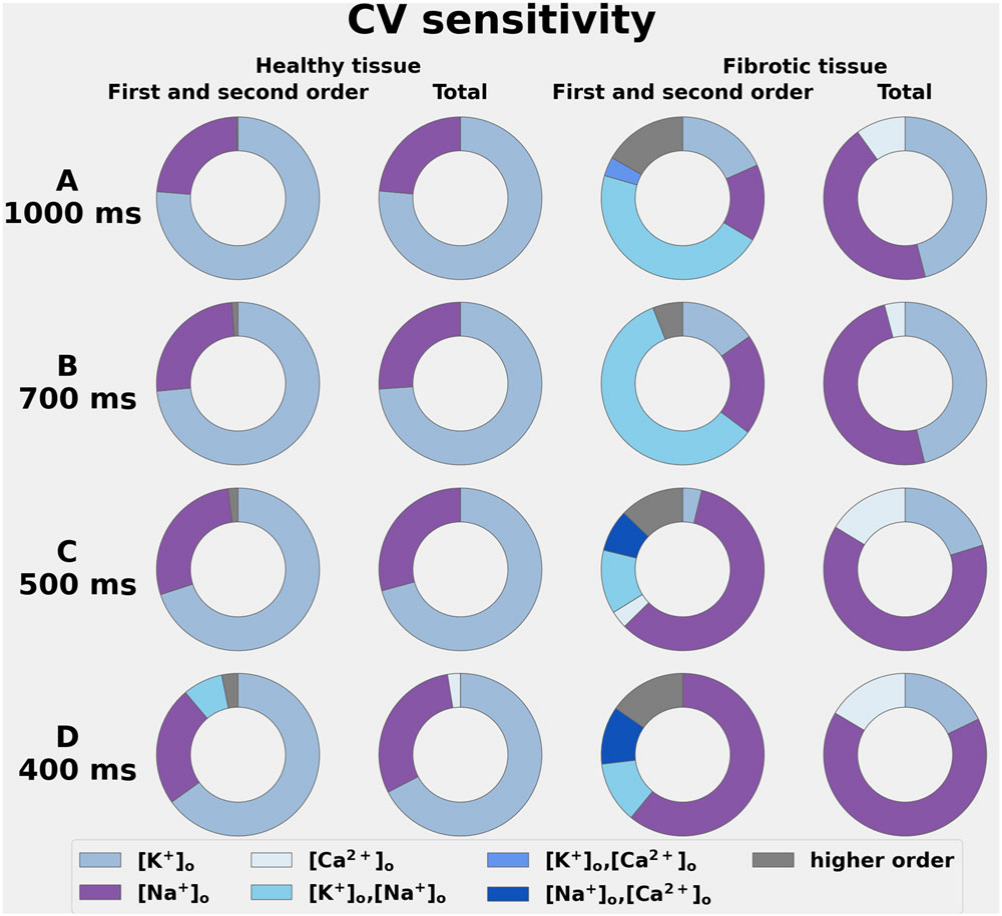
Demonstration of the sensitivity of the conduction velocity to selected alterations in plasma [Na^+^]_o,_ [K^+^]_o,_ and [Ca^2+^]_o_ studied at four different stimulus frequencies under baseline conditions and in the setting of fibrosis. The data in the two columns on the left show CV changes obtained from healthy tissue, while those on the right show these changes in the setting of atrial fibrosis. The colour coding denotes the changes in plasma electrolyte levels or their combinations that are shown at the bottom of this Figure. All data sets are expressed in terms of Sobol indices of the first order, second order, and total effects of the variance-based Global Sensitivity Analysis (GSA). At each cycle length, simulated regular pacing at one end of a one-dimensional homogeneous atrial tissue strand (1 cm long) was stimulated with a point source at one end. For each protocol, CV values were computed as the ratio between 0.5 and the difference between LAT measured at x = 0.25 cm and x = 0.75 cm. To complete Part 2 of this study, CV measurements were repeated for 120 different combinations of plasma electrolyte concentrations applied to both normal (baseline) and fibrotic atrial tissue strands. From the control or baseline data in the two left-hand columns of Rows (**A–D**) note that as expected, the predominant driver of conduction velocity values is [K^+^]_o_ derived through its effect on RMP; although alterations in [Na^+^]_o_ also have small effects (see Discussion). In contrast, in the setting of atrial fibrosis the combined effect of changes in [Na^+^]_o_ and [K^+^]_o_ are noteworthy at the longer cycle lengths (Rows **A** and **B** right columns) while the effects of changes in [Na^+^]_o_ predominate (Rows **C** and **D** right-hand columns) at the two shorter cycle lengths. The changes in the setting of fibrosis are strongly modulated by the observed action potential lengthening (see Fig. 2) and related changes in the atrial refractory period (see Discussion).

In Part II of this study, responses obtained from simulated atrial tissue strands or trabeculae were utilized so that the sensitivity of conduction velocity (CV) to small variations in [K⁺]ₒ, [Na⁺]ₒ, and/or [Ca²⁺]ₒ could be assessed. These tests were done in each selected electrolyte level employed in Part I at cycle lengths of 400 ms, 500 ms, 700 ms, and 1000 ms as shown in Fig. 4. To achieve a limit cycle that accounts for the Na⁺/K⁺ pump, we performed 15 minutes of pre-pacing^28^. Again, these measurements were made under both baseline or healthy atrium conditions (the two left-hand columns in Fig. 4) and in simulated fibrotic atrial tissue conditions (the two right-hand columns in Fig. 4).

Simulated paced activation was applied at one end of a strand, 1cm-long, 100μm by 100μm homogeneous 3D atrial myocyte tissue slab (100μm resolution). Conduction velocity (CV) was evaluated from local activation times (LATs) measured at 25% and 75% along the length as described in detail in Methods. In the Supplementary. Fig. S-2 shows the changes in CV values when one electrolyte concentration at a time was varied, leaving the other electrolyte species at their baseline value.

Small alterations in [K^+^]_o_ produced significant changes in CV at all cycle lengths. This is an expected result since the atrial myocyte RMP is well known to significantly alter the extent of inactivation of the transient inward Na^+^ current (INa)^50^. This effect, and the fact that the exact value of the RMP sets the voltage difference between RMP and the threshold for initiation of the action potential, combine to significantly alter excitability, latency to firing, as well as macroscopic CV.

In addition, as shown in Row A through D of Fig. 4, alterations in [Na^+^]_o_ produce small variations in CV. This effect is perhaps more prominent in fibrotic atrial tissue.

**Fig. 5:**
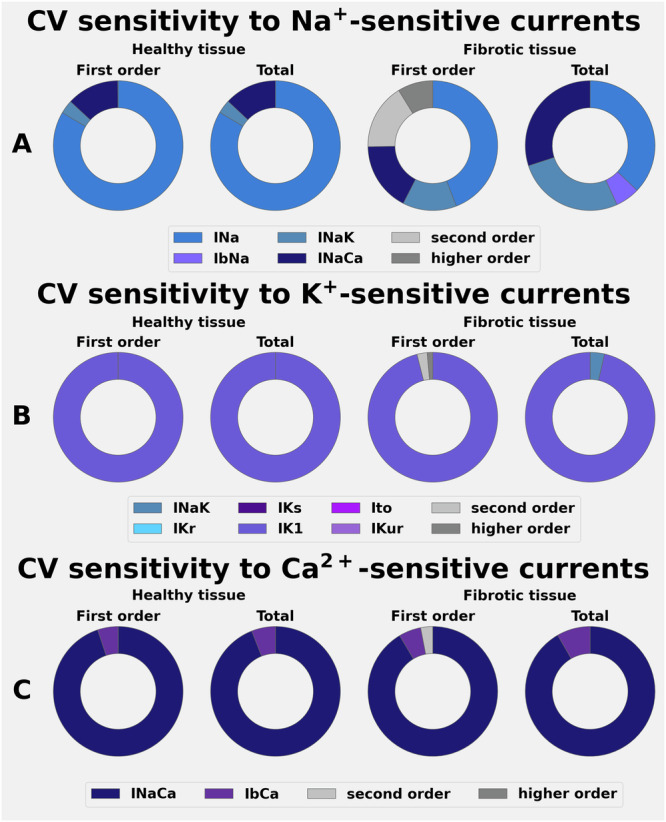
Illustration of the sensitivity of atrial conduction velocity to alterations in plasma electrolytes expressed in terms of ion channel or exchanger-mediated transmembrane ionic currents. GSA methodology was applied for each selected plasma electrolyte by varying each plasma level in the range ±25% for each of the transmembrane ionic currents that are listed below the Sobol indices plots in Rows (**A**–**C**). For example, one of these tests involved varying [Na^+^]_o_ in the mathematical expression for INaCa, while keeping [Na^+^]_o_ fixed at its reference value in the equations for all the other Na^+^-dependent currents. Additional description and interpretation of these data sets can be found in Results and Discussion.

These electrolyte-induced changes in CV can be further understood from an analysis of their sensitivity to electrolyte-induced changes in the main transmembrane ionic currents that are expected to be altered by each of the plasma electrolytes chosen for this study. Figure 5 shows which of these currents produces changes in CV when only one electrolyte concentration at a time is varied. The healthy tissue first and second, and total order effects (the two left-hand columns) are compared with those from fibrotic tissue. In healthy atrial tissue, the small CV changes observed following alterations in [Na^+^]_o_ are primarily driven by INa, with additional small contributions from the INaCa and INaK (the two left-hand columns in Row A). In contrast, in the setting of atrial fibrosis (the two right-hand columns in Row A), the contributions of both INaCa and INaK play a more prominent role in CV changes (see Discussion). Figure 5 Row B shows an analogous assessment of the effects of changes in [K⁺]ₒ on CV in the 1-D or simulated atrial strand/trabeculum. It is apparent that these electrolyte-induced changes modulate CV, primarily by changing the background K^+^ current IK1. It is well-known that the atrial myocyte RMP is strongly modulated by this current^28^. When plasma Ca^2+^ was altered (Fig. 4) CV was not changed significantly either under baseline conditions (left columns) or in the setting of fibrosis (right columns). This is not surprising^51^ since the selected range of changes in [Ca^2+^]_o_ was very small (approximately ±0.5 mM; see Discussion).

### Simulations of responses to paced activation of each human left atrium data set

In Part III of this study, the effects of selected plasma electrolyte changes were assessed in each of the 100 human left atrial data sets that were the focus of this study. Given the very substantial computational requirements of this work, we adopted the following study design. In total, for each atrial preparation, 30 combinations of plasma electrolyte (listed in the Supplementary Table S2) concentrations were studied. For each, the relative difference in quantities of interest (QoI) were computed and compared with the patient-specific reference or baseline levels in each of the 100 human left atrial digital twin data sets. In total, therefore, 3100 activation patterns were analysed to determine patient-specific mean values and variance levels for both APD and CV.

With this data set in hand, for each of the 100 patient-based data sets, we trained a Gaussian process emulator that mapped selected [K^+^]_o_, [Na^+^]_o_, and [Ca^2+^]_o_ levels to each QoI. To complete this analysis, the same GSA procedure that was used in Parts I and II of this study was applied. Although this approach was robust and proved to be broadly applicable, we note that 7 of these simulations (<0.3%), obtained from 4 different patient-based atrial anatomy data sets, developed a self-sustaining arrhythmia in response to a single pacing stimulus. These results were excluded from this analysis. Interestingly, all these excluded data sets (corresponding to entries 22 and 27 in Table S-2) were generated in the settings of hypokalaemia ([K^+^]_o_ between 3 and 3.3 mM) in conjunction with severe hyponatremia ([Na^+^]_o_ between 108 and 111 mM).

**Fig. 6:**
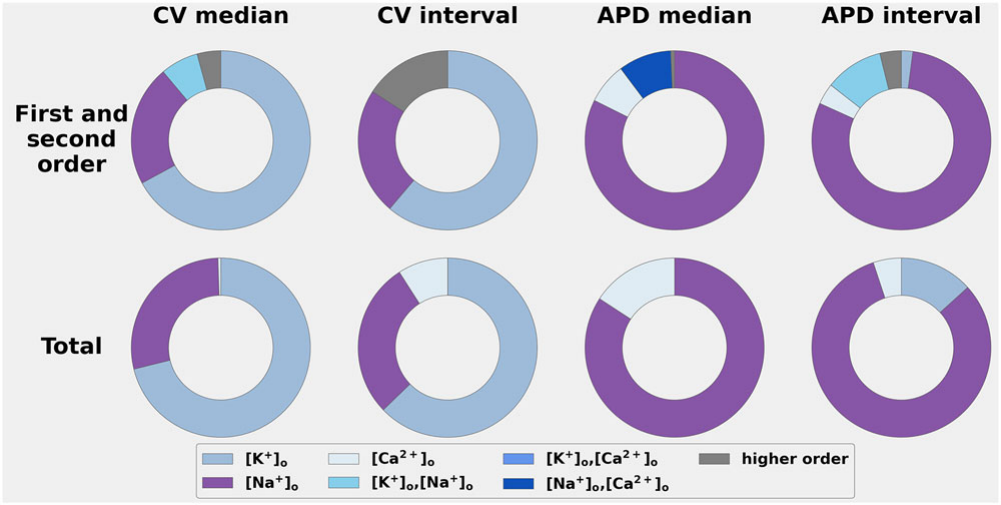
Demonstration of the effects of plasma electrolyte levels on CV and APD values derived from paced simulation of all 100 human left atrial samples. Global sensitivity of the median CV; inter-percentile CV, median APD, and inter-percentile CV to variations in [Na^+^]_o_, [Ca^2+^]_o_, and [K^+^]_o_ data sets are shown. This GSA analysis yielded patterns of results that were very similar to those that were obtained from analogous studies done using single atrial myocytes or strands of atrial tissue. Accordingly, this data set provides baseline information against which the manoeuvres for inducing atrial rhythm disturbances when plasma electrolytes are changed can be compared.

Figure 6 is constructed from the GSA output data consisting of the CV and APD values obtained from each of the 100 patient-specific data sets. As shown in the 2 left-hand columns, the CV values are very sensitive to variations in [K^+^]_o_, and also exhibit moderate sensitivity to variations in [Na^+^]_o_. In contrast, the electrolyte-induced changes in APD (the 2 right-hand columns) appear to be dominated by [Na^+^]_o_ changes. In summary, therefore, these findings, obtained from measurements in the entire (*n* = 100) left atrial data are very similar to the results computed using either a single myocyte (Fig. 3), or an atrial tissue strand (Fig. 4). Importantly, however, the data in Fig. 6 serve as an essential control for the final part of this study; and further analysis of plasma electrolyte-induced changes in induced arrhythmias in this digital twin data set.

### Simulations of patient-specific induced human atrial arrhythmias following plasma electrolyte changes

To complete Part III of this study, some aspects of the ways in which changes in plasma electrolytes can induce or sustain atrial arrhythmias were assessed for each of the 100 ‘virtual patients‘. To do this, atrial arrhythmias were initiated by: i) pacing near the left atrial appendage (LAA); ii) pacing on the atrial roof (AR); and iii) imposing a four-spiral-wave initial state with opposing chirality^43^, computed using UACs^26^ (see Methods).

For each of these simulated arrhythmias, binary (True/False) endpoints were assigned for successfully induced arrhythmias: i) following burst-pacing on the LAA; ii) following burst-pacing on the AR; iii) following either of these two burst-pacing; and iv) failure to sustain an arrhythmia for at least five seconds, denoted as ‘terminated‘. Results are summarised in the Supplementary. Table S-3; each column represents an endpoint.

Given that global sensitivity analysis is unsuitable for studies that have binary outcomes, a random forest classifier was instead fit to each of the 100 human atrial data sets, when each endpoint included at least two samples for each of the 2 outcomes. Table S-3 illustrates, for each endpoint, the number of individual atrial data sets that presented at least two samples for each outcome (Row B) and also shows the corresponding R^2^ scores (coefficient of determination) (mean, ±standard deviation, and min-max, Row C). These results therefore, link the input (plasma electrolyte concentrations) to the outcomes on a case-by-case basis. Stated differently, this pattern of results provides a basis for relating the selected plasma electrolyte concentrations to our chosen atrial arrhythmia outcomes and doing so on a patient-specific basis.

To complete this analysis and attempt to further highlight the interactive, and in some cases, cumulative proarrhythmic effects of plasma selected electrolyte levels in the presence of simulated atrial fibrosis, a classifier was trained for each of the four endpoints described previously again, using the entire human atrial data set (*n* = 100) and augmenting these input features with the patient-specific amount of fibrosis and the relative left atrial surface area. Table S-3 (Row D) summarises these R^2^ scores.

To extend and complete the analyses, a feature importance permutation analysis (FIP)^52^ was combined with Shapley analysis (SHAP)^44^ to gain insights into how extracellular plasma electrolyte concentrations, the fibrosis burden, and the left atrial surface area may affect the induction and/or sustainability of human left atrial arrhythmias.

**Fig. 7:**
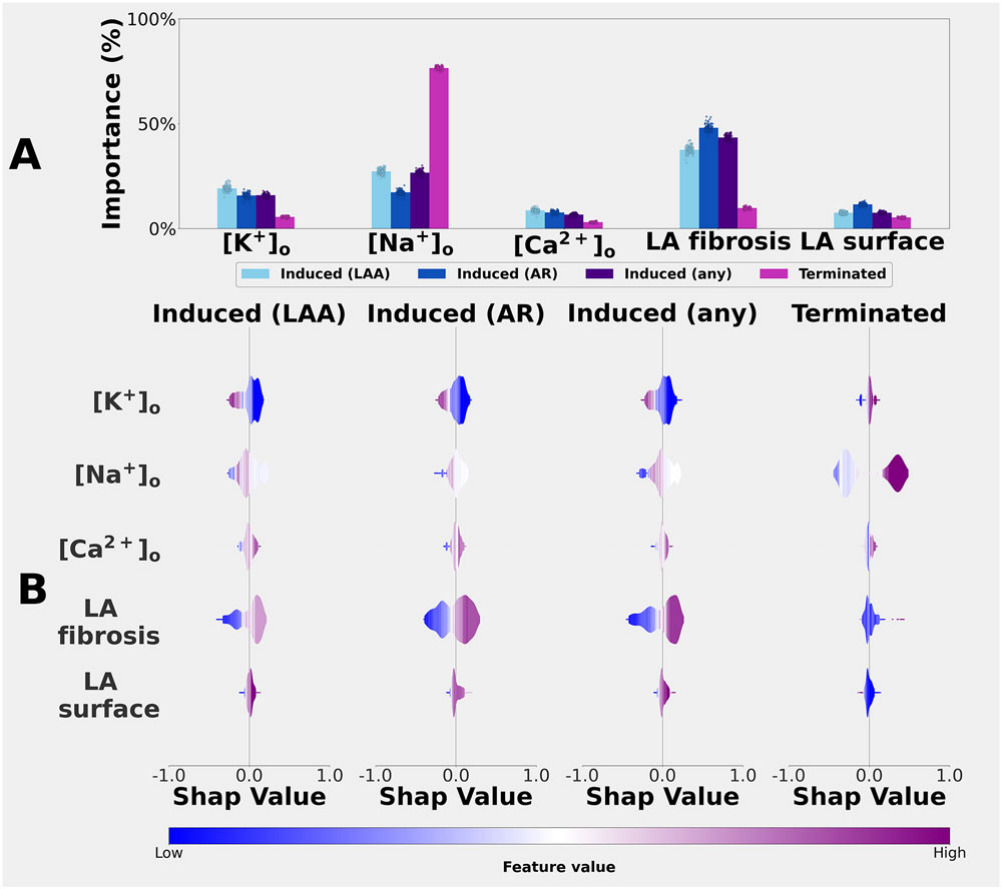
Histograms of the feature importance and violin plots of Shapley indices over the entire cohort of selected changes in [K^+^]_o_, [Na^+^]_o_, and [Ca^2+^]_o_ concentrations, in conjunction with the fibrosis amount and atrial surface area. Feature importance (**A**) was computed by permuting *n* = 100 times the values in the input feature space (each data point in the same plot represents a permutation). For each feature, the mean importance and the confidence interval (error bars) corresponding to one standard deviation were plotted. We considered the following endpoints: arrhythmia induced by a burst pacing in the proximity of the left atrial appendage (LAA), arrhythmia induced by a burst pacing in the proximity of the left atrial roof (AR), arrhythmia induced by a burst pacing, either on LAA or on AR (any), and arrhythmia terminated within 5 s from initiation. Note that induced arrhythmias termination strongly depended on [Na^+^]_o_, while no single effect was determinant for the initiation. Shapley indices (**B**) show that Hypokalaemia produced a pro-arrhythmic substrate, although this alteration alone was not sufficient to induce arrhythmias.

The same analysis confirmed that [Na^+^]_o_, and particularly hyponatremia, figured prominently in determining how long the induced arrhythmia lasted. In Fig. 7 Panel A, this parameter is denoted ‘Terminated’. Interestingly, these findings also reveal that adding a fibrosis burden has little if any effect on the sustainability of an induced arrhythmia.

Thus, judged by a case-by-case analysis (Supplementary Fig. S-9), [Na^+^]_o_ was important in determining termination; that is, hyponatremia favoured or enhanced arrhythmia sustainability. Although no single plasma electrolyte changes strongly modulated arrhythmia initiation, this SHAP analysis suggested that there may be an inverse relationship between [K^+^]_o_ and human atrial arrhythmia inducibility (see Discussion).

Figure 7 consists of FIP histograms (Panel A) and SHAP violin plots (Panel B) based on the results generated by each classifier after being trained by using the entire human atrial data set of simulations and with augmented input features. We note that inducibility was positively and quite strongly associated with the fibrosis amount. A second effect, although less prominent, was that lowering [K^+^]_o_ appeared to increase the likelihood of inducing an arrhythmia. In contrast, neither changes in [Ca^2+^]_o_ nor differences in left atrial surface area had any significant influence on the induction or sustainability/termination of these simulated atrial arrhythmias.

## Discussion

Our computations and sets of analyses have revealed that even relatively small changes in [Na⁺]ₒ ( ± 25%, or approximately 35 mM) consistently result in a tendency to a collapse the action potential (AP) plateau and therefore alter AP duration (APD) in single atrial myocytes, the atrial syncytia or strand, and entire human left atrial preparations (Figs. 2, 3 and 6). In addition, when simulating atrial fibrosis, a marked depolarisation of the resting membrane potential (RMP) was also observed. Global sensitivity analysis (GSA), as shown in Fig. 2, identified the electrogenic current generated by the Na^+^/Ca^2+^ exchanger, INaCa, as the primary driver of AP waveform changes. This may be somewhat surprising, because INa and L-type Ca²⁺ current typically govern excitability, and conduction velocity (CV), as well as AP plateau formation in human atria. The full explanation for these findings will require further investigation, including additional studies with other published models of the human atrial action potential that can accurately reproduce beat-to-beat [Ca^2+^]_i_^50,53^. Nevertheless, we suggest that the observed sensitivity of INaCa to changes in [Na⁺]ₒ is due to the resulting changes in its I-V relationship, particularly the generation of additional outward current at membrane potentials corresponding to the AP plateau (see Supplementary Fig. S-10).

Although the restricted range of [K⁺]ₒ levels—mildly hyperkalemic ( ~ 6.0 mM) to moderately hypokalemic ( ~ 3.0 mM)^54^—fall within clinically moderate ranges, both caused significant changes in RMP in all three human atrial ‘target‘ preparations. A change in RMP is well known to alter CV in human atrial preparations^54^, thus affecting the cardiac safety factor^55^ (see the supplement, section ‘Sensitivity of cardiac safety factor to electrolyte changes’). As noted in the Introduction, a previous study^19^ also employed a similarly modified CRN model of the human atrial action potential to assess the changes in plasma electrolytes that occur in the setting of haemodialysis for patients with significant renal failure. This comprehensive, multiscale analysis concluded that hypokalemia can render this compromised atrial substrate somewhat proarrhythmic.

The GSA analysis shown in Fig. 5 identified the inward rectifier background current (IK1)^56–58^ as the main contributor to alterations in RMP. It is well known that small [K^+^]_o_ changes can significantly alter the ion transfer or I-V relationship for this K^+^ conductance^59^. This well-studied phenomenon, often referred to as the ‘cross-over effect’, is due to there being K^+^ selective binding sites within the pore of these K^+^ channels and resulting ion-ion interactions, which can strongly alter this type of K^+^ channel conductance when even very small changes in [K^+^]_o_ occur^60^. The resulting shifts in RMP dramatically alter myocyte excitability and CV in the atrial strand as well as the entire left atrial preparations. In human atria^61^ and in a wide variety of other excitable tissues^62^, this occurs because the change in RMP influences Na^+^ channel inactivation, making them functionally unavailable to intrinsic or applied stimuli. This effect is especially pronounced in mammalian atria, where typical RMP in healthy tissues is in the -70 to -75 mV range, the steady-state inactivation relationship falls into this range of voltages^50^, and only approx. 10-20% of expressed Na^+^ channels are ‘available’ at -70mV.

Within the scope of this study, the variations in [Ca^2+^]_o_ (±0.45 mM from the control value of 1.8 mM) had negligible effects on RMP, APD, or CV values. This minimal impact was anticipated. Since these small changes in [Ca^2+^]_o_ would result in correspondingly minimal effects on Ca^2+^ conductances (generated by activated L-type Ca^2+^ channels). Moreover, the well-known ability of changes in [Ca^2+^]_o_ to alter membrane surface charges (the Frankenhaeuser/Hodgkin effect) and thus reduce electrostatic screening of voltage sensors by extracellular divalent cations^60^ would be minimal under these conditions. We acknowledge, however, that the baseline or control value of [Ca^2+^]_o_ (1.8 mM) was higher than normal clinical values (1.0 to 1.3 mM).

A secondary goal of this study was to evaluate how clinically relevant plasma electrolyte changes interact with spatially localised atrial fibrosis^63^. Our analysis compared APD and CV responses in healthy atrial tissue (Figs. 3, 4) to those obtained when using an atrial fibrosis model^42^, consisting of downregulating the transmembrane ion current conductances (as described in the Methods and in the Supplementary Table S-1). These electrophysiological modifications were applied to spatially localised patterns of fibrosis, identified using local IIR values and quantified into 6 different regions, characterised by the material properties summarised in the Supplementary Table S-1, for each of the 100 atrial tissue samples. Not surprisingly, incorporating this form of an atrial ‘fibrotic burden’ significantly altered the responses (APD and/or CV) in each atrial tissue strand or entire left atrium (Figs. 6 and 7). In summary, these simulations revealed an increased sensitivity to these two endpoints in response to even very small changes in either [Na^+^]_o_, [K^+^]_o_, or the combination of [Na^+^]_o_/[Ca^2+^]_o_, as shown in Figs. 5 and 6.

Although this analysis cannot provide a mechanistic explanation, it is known that electrotonic loading resulting from myocyte/fibroblast interactions^35,64^ has two consequences: i) it depolarises the atrial myocyte resting membrane potential (since RMP of the fibroblast population is −40 ± 10 mV); and ii) it alters the atrial AP waveform (see Figs. 2 and 5). This fibrosis-induced depolarisation of RMP reduces the excitability and increases the refractoriness in the heterogeneous syncytial substrate^50,62^, thus altering its ability to exhibit or maintain spontaneous rhythm disturbances. In Summary, while our approach to accounting for the electrophysiological effects of atrial fibrosis is somewhat simplistic, the main findings align with important clinical results, including patterns of induced atrial rhythm disturbances^25^.

In the final part of this study, sensitivity analyses and machine learning methods were applied in an attempt to assess whether the selected plasma electrolyte changes (alone or combined with patient-specific patterns of atrial fibrosis) could promote the initiation, or maintenance, of atrial arrhythmias^15,65^. Two endpoints were analysed: (i) the ease of initiating arrhythmias in response to three defined stimulus paradigms and (ii) arrhythmia sustainability, defined in terms of the arrhythmia lasting at least 5 seconds. Firing patterns during human arrhythmias are heterogeneous. It was noteworthy, therefore, that the atrial arrhythmia frequencies computed in this study^66^ had a mean frequency of 3.8 ± 1.1 Hz, a mean value which is compatible with published clinical findings^67^.

As shown in Fig. 7, changes in [Na^+^]_o_ produced the most prominent effects on atrial arrhythmia inducibility and sustainability. While the underlying ionic mechanisms have not been identified, the consistent patterns of arrhythmia inducibility and its sustainability, integrated across our 100 digital twin data sets, can be rationalised. In healthy atrial tissue, small changes in [Na^+^]_o_ produced: (i) significant APD variations, and (ii) a small depolarisation of RMP (Fig. 2). The combination of these altered excitability and refractoriness^50^, thus increasing the likelihood of arrhythmia initiation. Moreover, in fibrotic tissue, these electrolyte-induced proarrhythmic effects were more pronounced (Figs. 6 and 7), likely due to the fibrosis-induced significant depolarisation of RMP, and related alterations in excitability and refractoriness at the myocyte, tissue, and whole left atrial levels.

The arrhythmogenic role of [K^+^]_o_ is well established^8,28,68–70^. In both healthy and fibrotic atrial tissue, the selected ranges of [K^+^]_o_ changes (approx. ±1.5 mM) primarily modulated RMP, and thus the Na^+^ channel availability^50^. However, when RMP is depolarised significantly, the outward current (hyperpolarising effects) generated by an electrogeneic transport of the Na^+^/K^+^ pump (INaK) can also modulate excitability and perhaps inducibility^71^. Under these conditions, even minor shifts in [K^+^]_o_ can be proarrhythmic in both human atria^28^ and ventricles^70^. This classical effect is due to the associated flattening of the outward current region of the I-V relationship for IK1^60,72^. Although changes in [Ca^2+^]_o_ had no significant effects on APD or CV, minor effects on arrhythmia inducibility and sustainability are suggested (Fig. 7), warranting further investigation. Nonetheless, under the conditions of markedly reduced excitability (arising from a depolarised RMP and almost fully inactivated Na^+^ current)^50^, even small [Ca^2+^]_o_-induced changes in the voltage dependence for the activation or inactivation of the human atrial Na^+^ current could influence arrhythmia initiation or maintenance.

Nonetheless, several limitations in our study design and analysis need to be fully acknowledged.

1. Important components of the modified Courtemanche mathematical model for the human atrial action potential can now be updated. For example, the Courtemanche model provides only rudimentary formulations for replicating Ca^2+^-induced Ca^2+^ release. In part, this is because the reversal potential for the Ca^2+^ current (and all other currents) is fixed. In accordance with the suggestion of one Reviewer, we repeated the computations upon which Figs. 2, 3, and 4 were based after changing the formulation for ICaL to allow the reversal potential E_Ca_ to vary in response to changes in [Ca^2+^]_o_ (see Fig. S-11 and S-12 in the supplement). Furthermore, we repeated this set of calculations using the Coleman et al. (2013) model^73^ for the human atrial action potential. However, none of these changes significantly changed the pattern of results shown in Figs. 2 to 4. The most likely reason is that the small changes in [Ca^2+^]_o_ in this study altered the peak Ca^2+^ current insignificantly. Current atrial cell models may not capture all relevant physiology, and so our simulation results may be dependent on the specific modelling frameworks used; however, our results were consistent across two independent atrial cell models.
2. The functional importance of the current generated by INaCa could be reassessed after refining the ion transfer or current-voltage relationship for this exchanger-mediated electrogenic current. Specifically, the existing quasi-linear shape may be too simplistic and not able to accurately replicate important changes in the reversal potential for this important homeostatic mechanism.
3. An important part of our study was the characterisation of model-generated trains of induced atrial arrhythmias. We are aware that human atrial arrhythmias can show complex phenotypes and patterns^74^. Further validation of our patient-specific approach could involve distinguishing between early-onset/paroxysmal and chronic AF^65^. Since this information is available for each of the 100 atrial tissues that were the subject of this study, making this distinction could contribute to the emerging digital twin approaches towards personalised arrhythmia detection and management^75^. If this more detailed investigation was pursued, we also acknowledge that an additional set of calculations should be done in which the known electrophysiological changes in the atrial myocyte in the setting of arrhythmias were incorporated, would be valuable.
4. In this work, we studied only the isolated left atrium. We did not take into account fast conduction tissue such as Bachmann Bundle (BB) or Crista terminalis (CT). In addition, the marked heterogeneity of the right atrium, as well as different responses of its specialised conduction tissues, could produce a pro-arrhythmic substrate that contribute significantly to the overall human atrial substrate^73^.

In summary, this study demonstrates that personalised models of human atrial anatomy and electrophysiology can capture key functional and clinically relevant features, including sensitivity to small changes in plasma Na^+^ or K^+^ levels. By combining a multiscale computational approach (myocyte, atrial tissue, whole left atrium) with sensitivity analysis and some aspects of machine learning, our investigation draws attention to some of the most important underlying biophysical principles, while also yielding electrophysiological biomarkers that identify substrate excitability changes, including proarrhythmic tendencies for AF.

## Supplementary information

Supplementary information

Description of additional supplementary data

Supplementary Data 1

Supplementary Data 2

Supplementary Data 3

Supplementary Data 4

Supplementary Data 5

## Data Availability

All data required to reproduce the results presented here were previously published and are publicly available in the Zenodo repository (10.5281/zenodo.5801337)^76^. No restrictions exist on data availability.

## Code availability

Numerical simulations were performed with OpenCarp version 16.0 (https://opencarp.org/). Global sensitivity analysis of the biomarkers during paced activation has been conducted using the Gaussian process emulators implemented in the GPErks open-source framework (publicly available at: https://github.com/stelong/GPErks). and the Sobol global sensitivity analysis implemented in Salib v 1.4.7 (open source: https://salib.readthedocs.io/en/latest/). The analysis of the simulated arrhythmia was conducted with the random forest classifier and the feature permutation importance available in the open-source library scikit-learn version 1.2.2, while Shapley indices were computed using the open source library SHAP, version 0.42.1.
