# Supplementary material for: The effect of clinically relevant changes in extracellular electrolyte concentrations on human atrial arrhythmias
Source: Commun Med (Lond). 2025 Dec 2;6:7. doi: 10.1038/s43856-025-01260-4 (PMC12770435; doi:10.1038/s43856-025-01260-4)
Supplement: Supplementary file 1 — Supplementary information [file 43856_2025_1260_MOESM1_ESM.pdf]

The Effect of Clinically Relevant Changes in Extracellular Electrolyte Concentrations on Human Atrial Arrhythmias

Cesare Corrado[*1], Caroline H. Roney[2], Sanjiv M. Narayan[3], Wayne R. Giles[4], Steven A. Niederer[1]

[1]National Heart and Lung Institute, Imperial College London, London, United Kingdom

[2]School of Engineering and Materials Science, Queen Mary University of London, London, United Kingdom

[3]Department of Medicine and Cardiovascular Institute, Stanford University, Palo Alto, CA, USA

[4]Cumming School of Medicine, Department of Physiology & Pharmacology, University of Calgary, Calgary, Canada

[*]Corresponding Author: c.corrado@imperial.ac.uk

Supplementary methods

The modified Courtemanche (CRN) model of the human atrial action potential

The CRN model [1] is a robust, computationally efficient and quite widely used platform for simulation of basic electrophysiological features and responses to drugs of the so-called 'spike and dome' phenotype of the human atrial myocyte action potential [2–8] . Our group and others have effectively employed this model in studies having clinically relevant endpoints [9], for example. attempts to predict reoccurrence of AF after ablation procedures. However, the original or parent CRN model does <u>not</u> accurately reproduce the functionally important, physiological variations in either RMP or the APD when $[K^+]_o$ is altered [10]. We note that the original CRN model predicts a shorter APD when $[K^+]_o$ is decreased and a longer APD when $[K^+]_o$ is increased. In contrast, in vitro experiments show longer APD after decreasing $[K^+]_o$ and shorter APD after increasing $[K^+]_o$ [11–13]. Moreover, and importantly, <u>decreases</u> in $[K^+]_o$ often result in marked <u>depolarisation</u> of the myocyte RMP. These differences can be explained by the absence of mathematical terms that account for the well-known $[K^+]_o$ `sensing` or intrinsic regulation of IK1 and IKr, the two strongly inwardly rectifying K^+ conductances that are expressed in mammalian hearts. Corrected mathematical descriptors for IK1 [14] have been included in a number of more recent ventricular myocyte [15] and atrial myocyte models [16,17]. Accordingly, we modified the CRN model (CRN+) by incorporating the standard formulation for the GKr and GK1 conductances:

$$GKr = \overline{GKr}\sqrt{\frac{[K^+]_o}{5.4}} \qquad GK1 = \overline{GK1}\sqrt{\frac{[K^+]_o}{5.4}}$$

$\overline{GKr}$ and $\overline{GK1}$ are constant values. The reference value for $[K^+]_o$ is assigned as 5.4 mM. This reference $[K^+]_o$ concentration was employed under control conditions (at baseline) in the original CRN model, and it is utilized in most atrial and ventricular myocyte models [15–17] mainly because this is the $[K^+]_o$ used in Tyrode solution in most experimental settings. However, in human plasma $[K^+]_o$ values are close to 4.5 mM [18,19].

As shown in Figure S-1, a comparison of atrial myocyte APD values at a 1Hz stimulation rate reveals significant differences when the original CRN model (magenta line) and the modified CRN+ model (blue line) are compared. Moreover, these differences are variable depending upon the exact $[K^+]_o$ values that are employed. This range of plasma $[K^+]_o$ are the primary focus of this study. The sets of APD values generated by the CRN+ model agree quite closely with those published values for human atrial myocytes[10,11,20] .

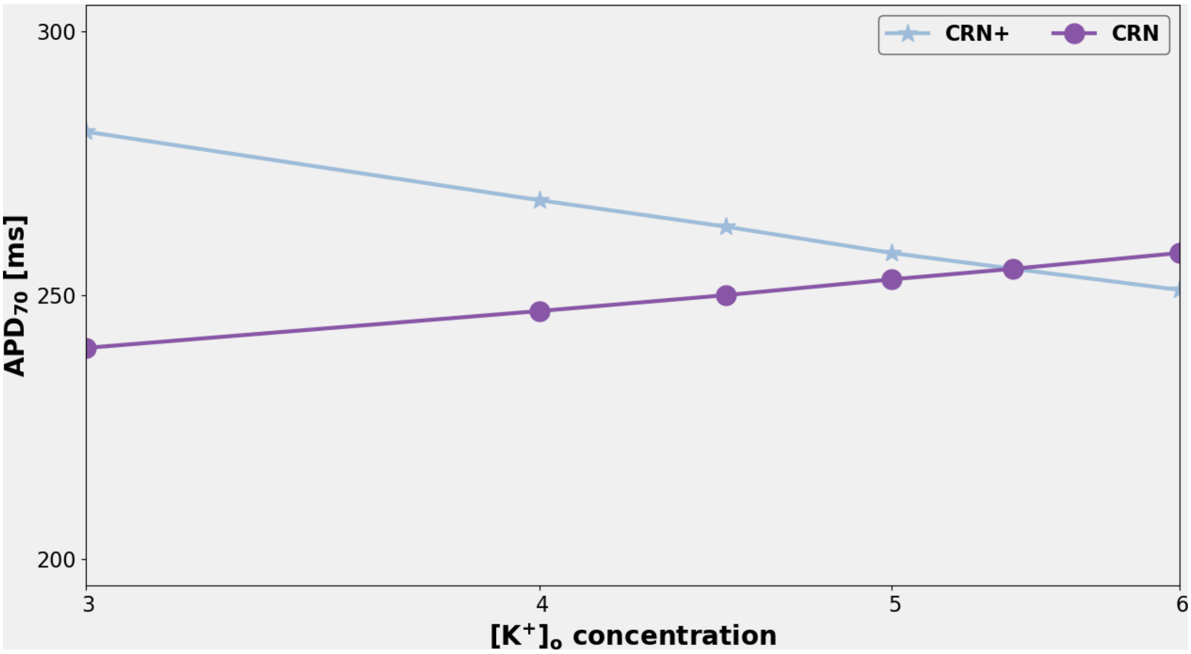

Figure S-1 ***Comparison of the durations of the atrial action potentials obtained using the original and the modified Courtemanche models.*** *Action potential duration (APD) values from the original Courtemanche model (CRN, magenta) are compared with the modified Courtemanche (CRN+, blue line) models, for a cycle length of 1000 ms and different $[K^+]_o$ concentrations. Note that the original CRN model does* *not* *reproduce the physiological variation of the APD for alterations of $[K^+]_o$* [10].

Human atrial tissue properties reflecting substrate heterogeneity

	IIR ≤ 0.9	0.9 < IIR ≤ 1.1	1.1 < IIR ≤ 1.22	1.22 < IIR ≤ 1.4	1.4 < IIR ≤ 1.6	IIR > 1.6
σ_{il} (S/m)	0.4	0.31	0.31	0.31	0.28	0.19
σ_{it} (S/m)	0.1	0.075	0.075	0.075	0.07	0.0475
GNa nS/pF	7.8	7.8	7.8	4.68	4.68	4.68
GK1 (nS/pF)	0.09	0.09	0.09	0.045	0.045	0.045
GCaL (nS/pF)	0.1238	0.1238	0.1238	0.0619	0.0619	0.0619

Table S-1 ***Human Atrial Tissue Electrophysiological Parameters scaled using MRI intensity ratios.*** *Based on the observed regional differences, MRI image intensity ratios (IIR) were calculated for each individual human atrial preparation. As shown in the top two rows, this provided the basis for scaling transverse and longitudinal conductivities in each atrial preparation. These IIR data sets were used as a basis for further subdividing the functional electrophysiological properties of each atrial preparation into six different phenotypes. The associated electrophysiological/biophysical descriptors were derived by assigning scaled values of the maximal conductances for: i) the fast transient inward Na^+ current; ii) the inwardly rectifying background K^+ current; and iii) the L-type Ca^{2+} current. In the second part of this study, the effects of atrial fibrosis, occurring in combination with plasma electrolyte changes, were simulated. As a first approximation to the electrophysiological effects of atrial fibrosis, in each patient-specific human atrial model the maximum conductances for INa (by 40%), IK1 (by 50%) and ICaL (by 50%)in the myocyte population were decreased by the amounts shown above.*

Matrix of individual plasma electrolyte levels

ID	$[K^+]_o$	$[Na^+]_o$	$[Ca^{2+}]_o$	ID	$[K^+]_o$	$[Na^+]_o$	$[Ca^{2+}]_o$
1	5.25	146.4	1.60	16	5.96	130.8	2.25
2	3.44	174.8	1.46	17	3.76	152.0	1.39
3	5.19	166.3	2.09	18	4.27	141.7	1.43
4	4.49	169.4	2.05	19	5.08	147.4	1.69
5	4.93	127.6	1.80	20	4.15	157.4	1.97
6	4.85	144.0	1.87	21	4.61	129.5	1.77
7	5.88	154.4	1.92	22	3.29	108.8	1.94
8	5.35	125.9	1.58	23	5.67	114.0	2.18
9	3.88	135.2	1.84	24	3.59	159.1	1.62
10	5.40	172.0	2.22	25	4.07	164.0	2.00
11	3.11	122.3	1.68	26	3.33	116.1	1.52
12	3.69	136.1	1.80	27	3.03	111.9	2.14
13	3.95	138.8	2.12	28	4.76	116.8	1.53
14	5.58	119.6	1.73	29	4.34	107.1	1.37
15	4.52	149.3	1.49	30	5.73	162.5	2.03

Table S-2 ***Plasma electrolyte concentrations values used in the numerical simulations in this study.*** *Here, the highlighted numbers under the two columns labelled ID, denote the 30 individual sets of plasma electrolyte levels that were evaluated so that their output could be compared with our selected control or baseline plasma electrolyte levels. For each labelled test or ID, the LAT procedure (see Methods) was used to assign the 3 individual plasma electrolyte levels.*

Variance-based global sensitivity analysis

The variance-based sensitivity analysis previously described in detail[21] were used in this study. Briefly, this approach expresses the output variance values as a sum of contributions due to variations in the input parameters and their interactions. First-order effects measure the direct impact on output when each input parameter is varied, while keeping the other input parameters fixed. Second and higher-order effects capture the interaction between two or more input variables, thus measuring their combined impact on the model output after two or more input parameters have been varied. The `total` effects express the overall influence of each selected input parameter on the output. That is, the total accounts for the first order effect and the effects due to interactions with all other variables. These effects are measured and illustrated using Sobol indices, which are defined as the ratio between the variance of the selected N-order effect and the total variance. Thus, the Sobol indices for each test sum up to 1.

In this study, Sobol indices for the first, second and total effects were obtained using the Salib library [22]. When up to three parameters(for example, 3 different plasma electrolyte levels) are selected for study, all these orders can be determined, using the following expression for the third-order effects:

$$S_{123} = 1 - \sum_{i=1}^{3} S_i - \sum_{i=1}^{3} \sum_{j=i+1}^{3} S_{ij}$$

Numerical integration was used to evaluate the Sobol indices. To obtain and evaluate first order, second-order and total effects, the algorithm implemented in Salib uses N*(2D+2) samples from the input parameter space, with D being the dimension of the input space and N being a user-defined hyperparameter. In this study, we chose N=1024. For each sample (electrolyte level) selected from the input parameter space, the CRN++ model output parameters (CV and APD) were obtained.

Electrophysiological simulations such as the ones that were essential for this study are computationally very demanding and sometimes even impractical. For this reason, simulating the electrophysiological features for all the samples required to compute the Sobol indices of interest is not feasible. To address this limitation, we used a surrogate model consisting of a simplified, computationally efficient approximation of the electrophysiology behaviour, expressed in terms of its quantities of interest (QoI). Specifically, we adopted the Gaussian process (GP) provided by GPErks, (available at https://pypi.org/project/GPErks).

The emulators were fitted using a data set of 31 samples. Once fitted, all the emulators presented an r2 score≥0.9. For each QoI (e.g. APD or CV), we evaluated the median of each Sobol index over the entire cohort of 100 individual human atrial data sets.

Criteria used to identify induced human atrial arrhythmias

For each of the three arrhythmia-inducing stimulus protocols previously described in Methods, successful induction and termination of the patterns of repetitive firing were detected as follows. The nodal average of the first temporal derivative of the transmembrane potential in each record was computed according to:

$$\overline{\left|\frac{dv}{dt}\right|}(k) = \frac{1}{N_{pt}}\sum_{j=1}^{N_{pt}}\left|\frac{v(k+1,j)-v(k,j)}{dt}\right|$$

v(k,j) denotes the transmembrane potential at time step k on vertex j. We determined the temporal index $k=k_{stop}$, corresponding to arrhythmia termination. as the first `occurrence` that satisfies the following two inequalities:

$$\overline{\left|\frac{dv}{dt}\right|}(k) < 10^{-4}$$

$$\left|\overline{\left|\frac{dv}{dt}\right|}(k+1) - \overline{\left|\frac{dv}{dt}\right|}(k)\right| < 10^{-4}.$$

Valid detection of the start of each such arrhythmia was classified as being successfully `induced` if k_{stop} criteria were not met for data sets generated by either of the stimulus protocols 1 or 2 that have been described in Methods. In addition, each induced arrhythmia was classified as `terminated` if k_{stop} values existed for all three of the arrhythmia-inducing protocols.

Induced human atrial arrhythmia classifiers

For each endpoint (induced/terminated atrial arrhythmias), the following classifiers were trained using *scikit-learn* [23]:

- logistic regression with polynomial features
- random forest
- gradient boost

The hyperparameters for each classifier were tuned using a grid-search algorithm and a repeated stratified k-fold cross-validation. Next, each classifier was trained using the optimal hyperparameters applied to the entire data set (n=100). The following hyperparameters were optimised:

i. For logistic regression: the degree p of the polynomial features (maximum degree =3) and the regularisation parameter C (20 values evenly spaced on a log scale, between 10^{-5} and 10^{5}).
ii. For random forest: the number of estimators (between 50 and 250), the classifier criterion (gini, entropy or log_loss), the maximum depth (between 3 and 6), the minimum number of samples per leaf (between 3 and 6), and the minimum number of samples that produce a split (6 to 8).
iii. For gradient boost: the number of estimators (between 50 and 250) the classifier loss (log_loss or exponential), the classifier criterion (friedman_mse or squared error), the learning rate (4 values evenly spaced in a log scale, between 10^{-2} and 10^{-5}), the maximum depth (between 3 and 6), the minimum number of samples per leaf (between 3 and 6), and the minimum number of samples that produce a split (6 to 8).

Whenever possible, we used the option "balance" to address the problem of the skewness of the data set. We did not apply our training procedure in situations where either output class comprised of less than two samples.

Supplementary results

Variations of the conduction velocity due to alterations in electrolyte plasma concentrations

Figure S-2 shows the patterns of change in CV when only one plasma electrolyte species is altered per test computation, leaving the other electrolyte values at baseline concentrations. CV values are plotted for the healthy atrial tissue (blue line) and in the settings of fibrosis (magenta line). Four different stimulus cycle lengths were considered: 1000 ms (row A), 700 ms (row B), 500 ms (row C), and 400 ms (row D).

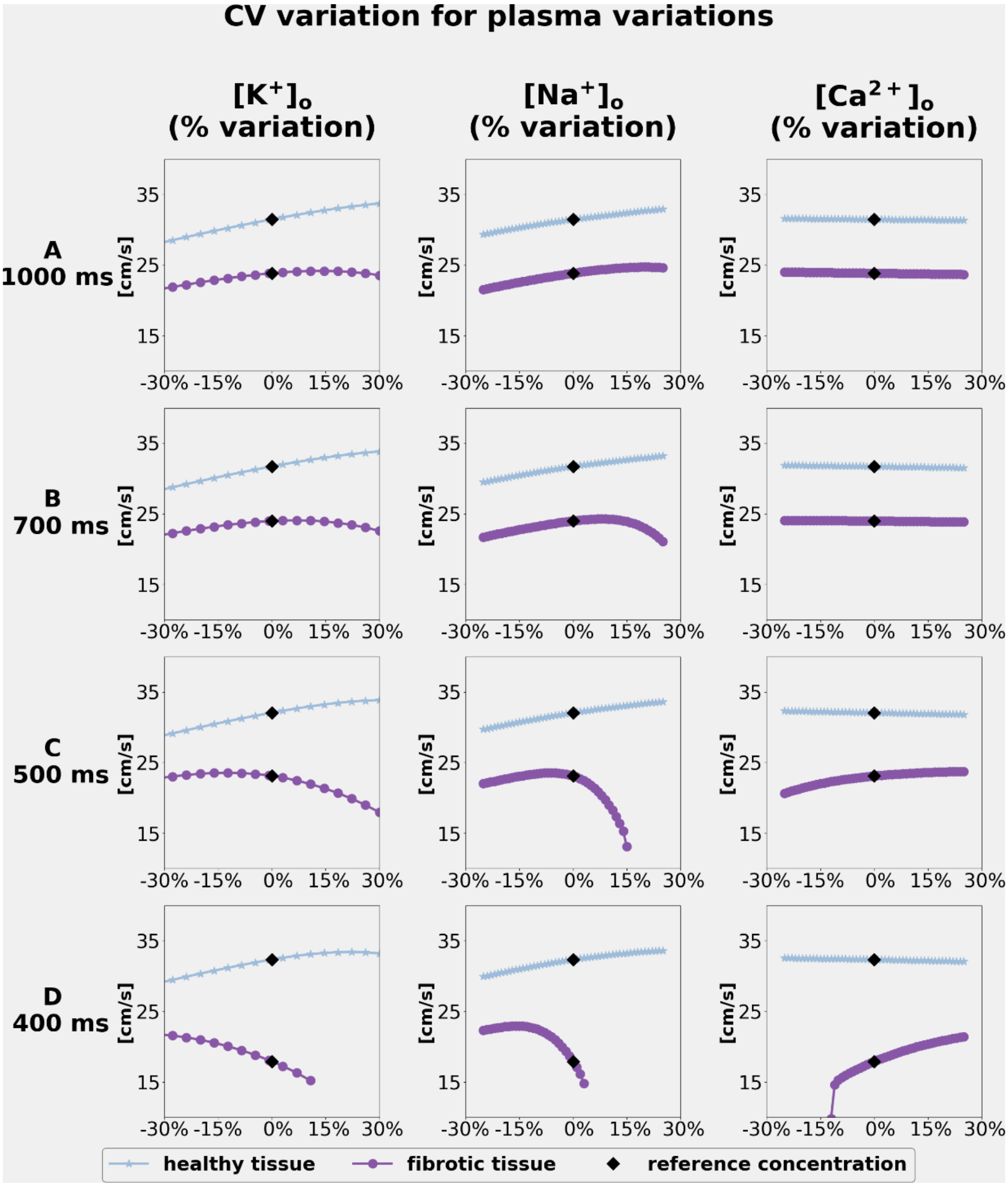

Figure S-2 ***Patterns of change in CV vs plasma electrolyte concentrations****. Each CV value was obtained by varying one of the three electrolyte levels at a time (from the baseline condition). Experiments were done for the healthy tissue (blue line) and in the setting of fibrosis (magenta line). Four different stimulus cycle lengths were considered: 1000 ms (row A), 700 ms (row B), 500 ms (row C), and 400 ms (row D). Variations in $[Ca^{2+}]_o$ (right column) have a*

negligible effect on the conduction velocity in the healthy tissue, while the fibrotic tissue presents a weak dependency at a cycle length of 500 ms (row C). Alterations in $[Na^+]_o$ (centre column) produce mild to strong CV variations in the settings of fibrosis, with a tendency to change inversely in response to the cycle length (rows B and C). The selected changes in $[K^+]_o$ can alter CV and the effect are more prominent in the setting of fibrosis (See results and Discussion)

Comparisons of human atrial and ventricular myocyte action potential responses to selected electrolyte changes

To illustrate key differences between human atrial and ventricular myocyte action potential models in terms of their responses to changes in plasma electrolytes, we applied the 0-D and 1-D analyses described in Methods. The standard Tomek et al[24] human ventricular myocyte action potential model was selected based mainly on its documented ability to accurately reproduce the dependency of the APD on small (± 25%) changes in $[K^+]_o$.

This pattern of results shown in Figure S-3 provide a mechanical comparison of the responses of this ventricular model to those that we obtained using the CRN+ model of the human atrial action potential. As shown in Panel A changes in $[K^+]_o$ produce significant alterations of both APD and RMP. It is well known that this occurs mainly because of the background inwardly rectifying K^+ current IK1 being large in all ventricular myocytes [25–27]. Analogous (±30%) variations in $[Na^+]_o$ altered both the plateau and the repolarisation phases of the ventricular action potential. In contrast, (+/- 30%) changes in $[Ca^{2+}]_o$ resulted in only small alterations in the waveform of the human ventricular action potential.

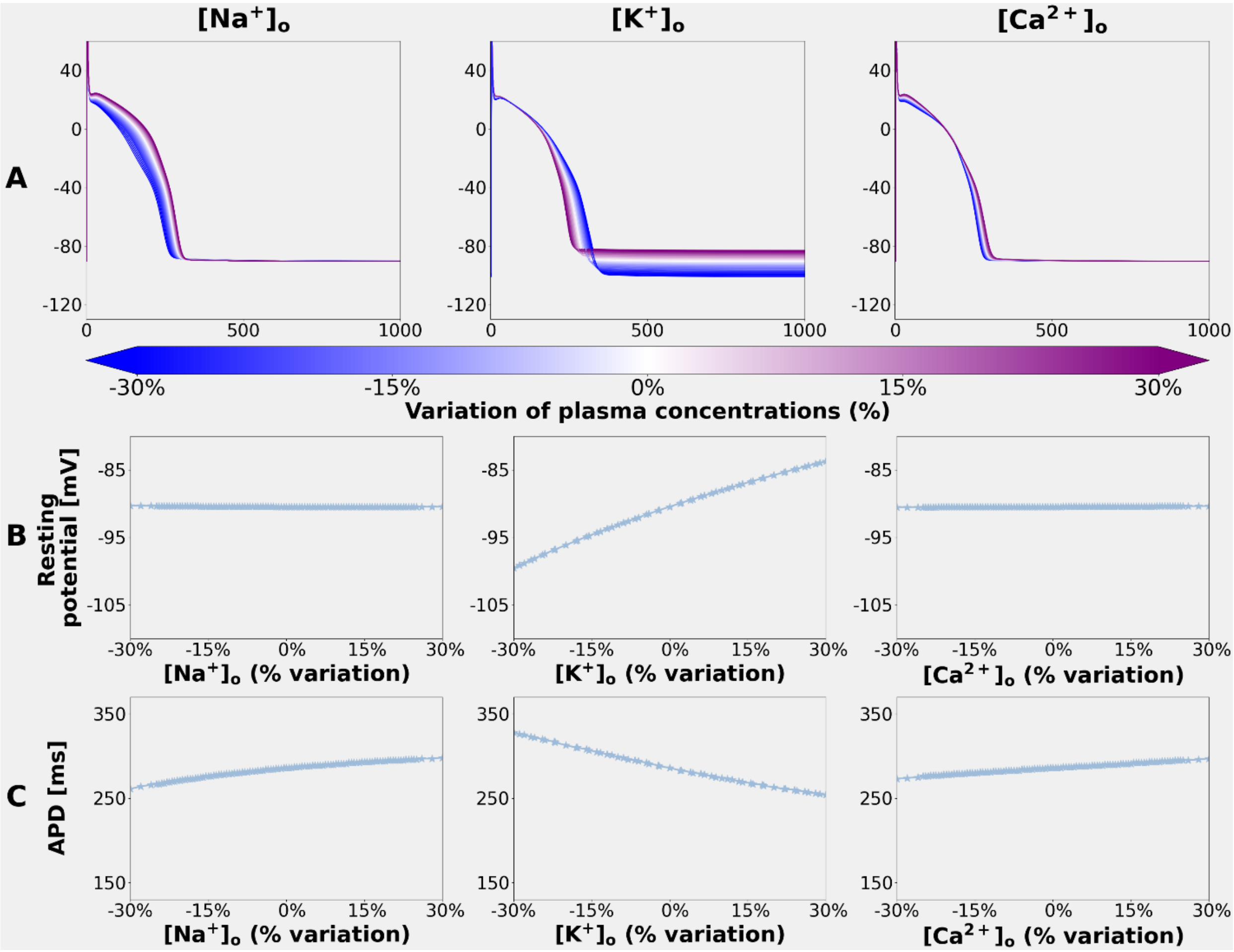

Figure S-3 ***Effects of selected alterations in plasma electrolytes on the human ventricular myocyte resting membrane potential and action potential waveforms.*** *Row* ***2A*** *shows the effects of ± 30% alterations in $[Na^+]_o$ (left), $[K^+]_o$ (centre), and $[Ca^{2+}]$ (right) on the action potential waveform and RMP illustrated using the colour coding shown at the bottom for the extent of these changes. The selected changes in $[Ca^{2+}]_o$ on the action potential waveform are negligible and do not alter RMP (Row* ***2B****, right), nor the action potential duration at 90% of the repolarisation (APD_{90}, Row* ***2C****, right). Variations in $[Na^+]_o$ alter the plateau height (phase 2) and the beginning of phase 3 (repolarisation) leading to small changes in the APD_{90} (Row* ***2C****, left) but have no significant effects on the RMP (Row 2****B****, left). In contrast, and importantly, ± 30% changes in $[K^+]_o$ have a significant effect on the RMP (Row* ***2B****, centre) and also modulate the end of phase 3, or final repolarisation of the AP, leading to significant alterations in APD_{90} (Row 2****C****, centre).*

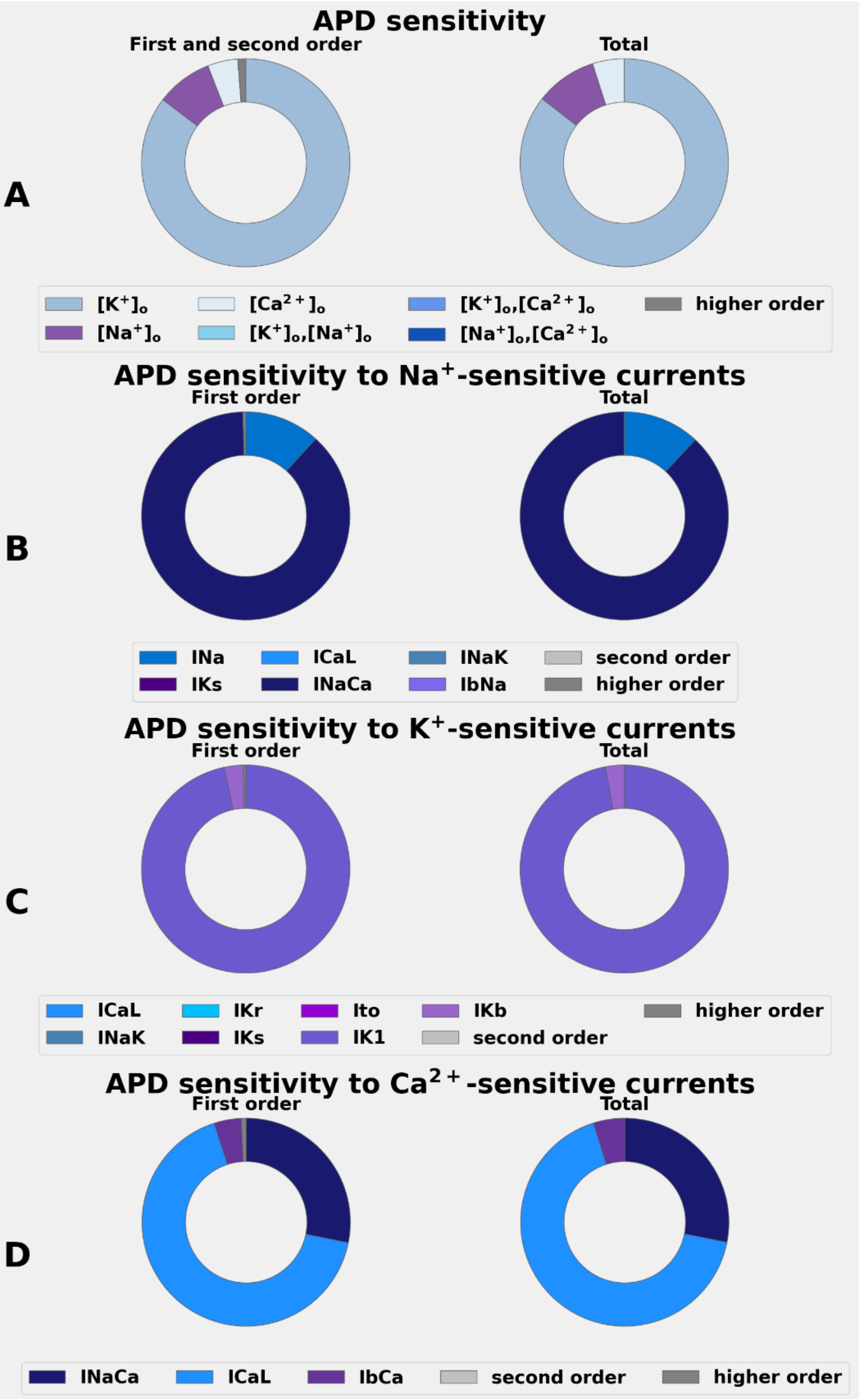

Figure S-4 ***Demonstration of the sensitivity of the human ventricular APD and underlying Na^+, K^+ and Ca^{2+} currents in response to changes in plasma electrolytes.*** *Row A illustrates the relative sensitivity of the human ventricular APD_{90} to ±30% changes in the 3 selected plasma electrolytes (as well as combined changes in some of these e.g., $[Na^+]_o$ $[Ca^{2+}]_o$). Each of these manoeuvres was assessed one at a time. As expected, and is shown in Row A, the $[K^+]_o$ changes produce prominent effects on APD. The APD is also altered by changes in $[Na^+]_o$. The findings from this sensitivity analysis shown in Rows B, C and D identify the changes in the transmembrane ionic currents during the action potential that are responsible for the observed APD alterations. Ventricular APD is strongly sensitive to variations in $[K^+]_o$ due to the IK1 current. The small sensitivity to $[Na^+]_o$ is generated mainly by the Na^+/Ca^{2+} exchanger current, and the less prominent sensitivity to $[Ca^{2+}]_o$ arises from ICaL.*

Figure S-4 consists of a series of plots depicting the results of GSA-based sensitivity analysis. Rows A through D illustrate the combined first and second order sensitivity (left column) of the 6 selected variables as well as the total sensitivity analysis (right column). As shown in Row A, when sensitivity to APD changes is selected, alterations in $[K^+]_o$ predominate. The Sobol plot summaries in Row C add significantly to this analysis by illustrating that the effects on APD in Row A can be accounted for mainly by the observed changes in the background K^+ current, IK1, determined by both first order and total variability analyses. Results of this analysis of the effects of changes in $[Na^+]_o$ shown in Row B emphasize the importance of the changes in the electrogenic current generated by the Na^+/Ca^{2+} exchanger with lesser but notable effects due to changes in INa. The pooled results in Row D show that when $[Ca^{2+}]_o$ is altered, the corresponding changes in APD are mainly due to alterations in the L-type Ca2+ current (as expected).

The next set of computations were done using simulated human ventricular tissue strip or trabeculum driven by the action potential model published by Tomek et al [24]. The main goal of this part of our study was to identify the predominant effects on conduction CV due to the selected changes in the 3 plasma electrolytes, and/or their combinations e.g., $[Na^+]_o$ and $[Ca^{2+}]_o$; or $[Na^+]_o$ and $[K^+]_o$. Each of these changes was studied at the 4 different cycle lengths shown in Figure S-5. This somewhat complex pattern of results reveals 2 significant trends at all cycle lengths: i) changes in $[Na^+]_o$ significantly and consistently alters CV, and ii) the same predominant effect is observed when $[K^+]_o$ is altered. Although the exact underlying causes for this sensitivity of CV to these changes requires further study, the well-known $[K^+]_o$-induced change in the RMP (see discussion) and resulting alterations in the availability or extent of inactivation of INa are certainly of importance.

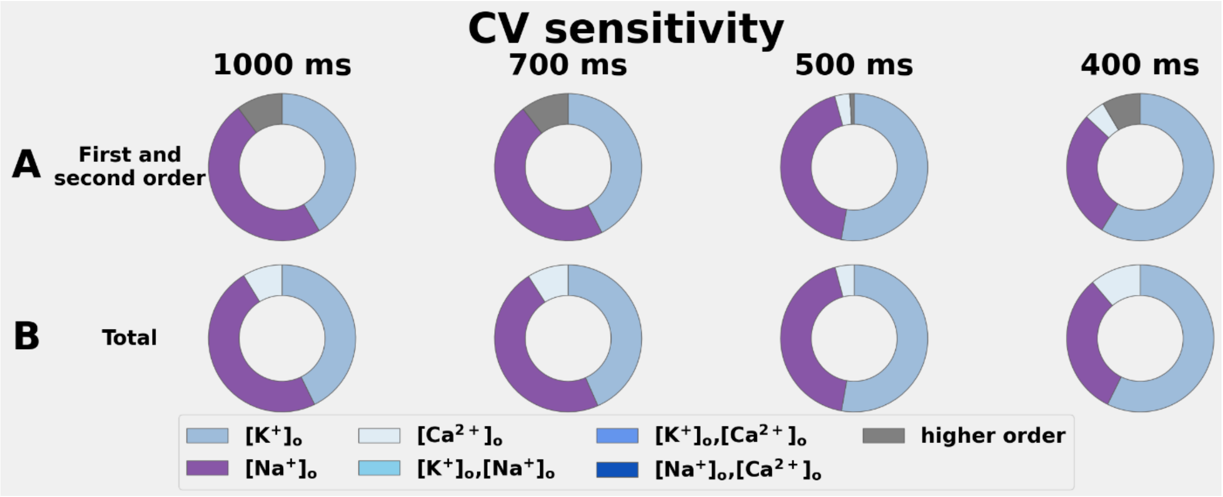

Figure S-5 ***Demonstration of the sensitivity of the conduction velocity to small selected alterations in plasma $[Na^+]_o$, $[K^+]_o$ and $[Ca^{2+}]_o$ obtained at four different stimulus frequencies using a human ventricular myocyte action potential model.*** *These changes were studied at four different stimulus train cycle lengths shown at the top of each column. See text for further explanation.*

The summarised data in Figure S-6 illustrate the effects on CV values of the same variations in plasma $[Na^+]_o$ or $[K^+]_o$, again measured using *in silico* human ventricular myocytes. In this case CV sensitivity has been analysed in terms of the six named ionic currents that contribute to the action potential and/or the RMP. The results in Row A of this Figure S-6 show that in the ventricular myocyte preparation when $[Na^+]_o$ is changed, CV is mainly sensitive to or modulated by the transmembrane current, INa. The results in Row B illustrate a more complex set of

responses to changes in $[K^+]_o$. However, these effects on the inwardly rectifying background K^+ current, IK1, predominates very likely as a result of its ability to change the resting potential and thus alter the availability of INa, ICaL, as well as INaCa.

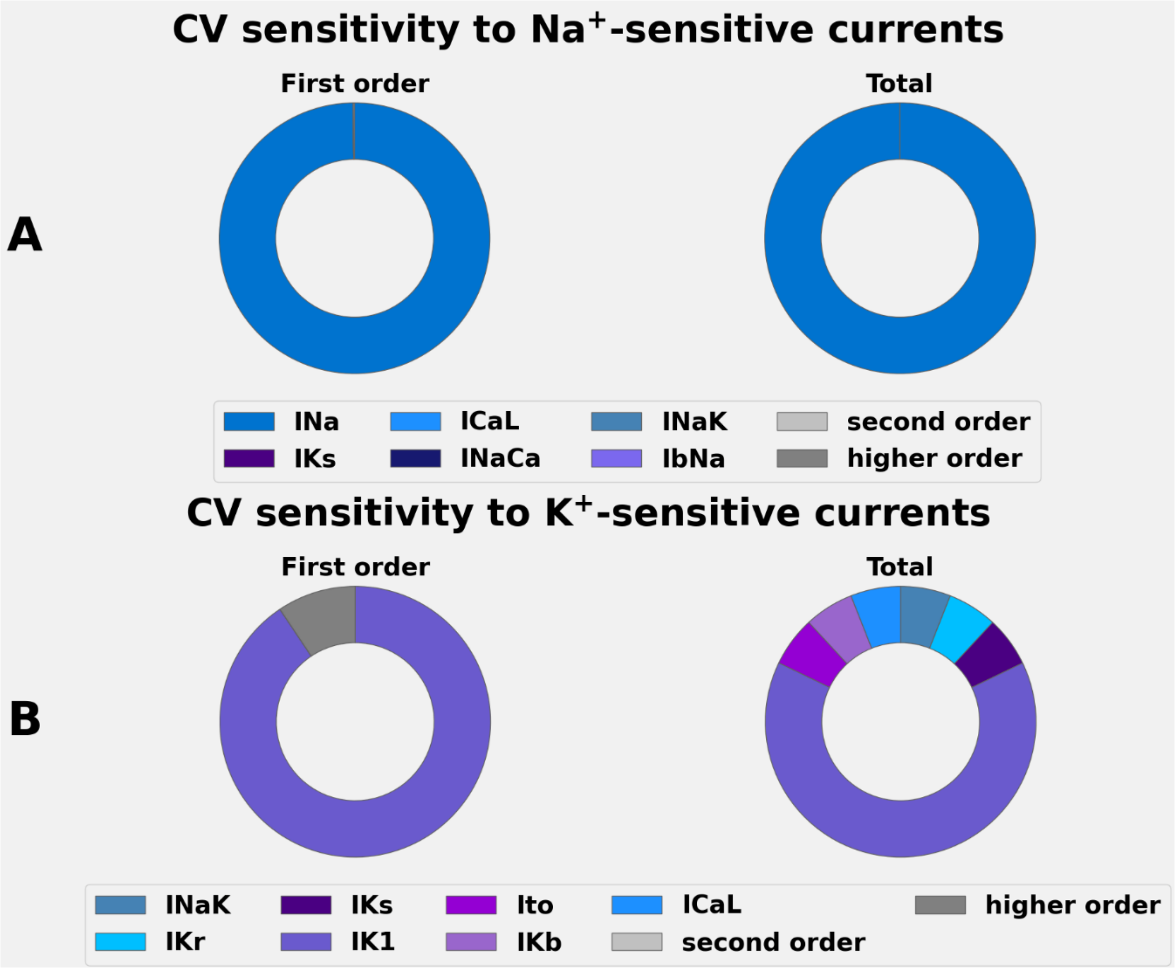

Figure S-6 ***Illustration of the effects of changing the plasma electrolytes $[Na^+]_o$ or $[K^+]_o$ on human ventricular conduction velocity expressed in terms of the individual transmembrane ionic currents that are listed below in Rows A and B.*** *For each manoeuvre, the GSA parameters produced by varying the electrolyte concentration of interest is shown. For example, $[Na^+]_o$ was varied in the mathematical expression for INaCa while keeping $[Na^+]_o$ fixed to its reference value in all the other equations for $[Na^+]_o$-dependent currents. Note from Row A that CV is altered mainly through changes in the fast inward sodium current, INa. As shown in Row B changes in $[K^+]_o$ modulate CV mainly by altering the inward rectifier K^+ current IK1 and related changes in RMP. See text for further explanation.*

Effects of severe hypo- or hyperkalaemia in human atria

Well-known clinical settings can result in changes in plasma $[K^+]_o$ levels that are much larger than ±25% from normal, 4.5 mM. These clinical settings include but are not limited to post-heart surgery recovery time periods and haemodialysis[28]. In both situations, $[K^+]_o$ can initially be remarkably high but also can be reduced to hypokalemic values in response to effective clinical treatment. These changes in $[K^+]_o$ are known to contribute to a proarrhythmic atrial substrate [28]. In addition, prolonged periods of high heart rates, such as those that occur in endurance exercise, can significantly increase $[K^+]_o$ [29].

Accordingly, we have tested our modified atrial myocyte action potential model (CRN++) after setting $[K^+]_o$ to fixed values in the range of 1.35 to 10 mM at a cycle length of 1000 ms. Figure S-7 summarises the changes in APD (left) and CV (right) in response to these manoeuvres. Results obtained using the CRN++ model are illustrated in blue and an analogous data set obtained after introducing simulated atrial fibrosis is shown in magenta. The monotonic changes (progressive shortening) of the APD are a conventional pattern of results. The biphasic effect of hyperkalemia on CV, that are first observed with $[K^+]_o$ levels larger than approximately 8 mM in normal atrial tissue [12], and approximately 6 mM in fibrotic atrial tissue have also been reported previously.

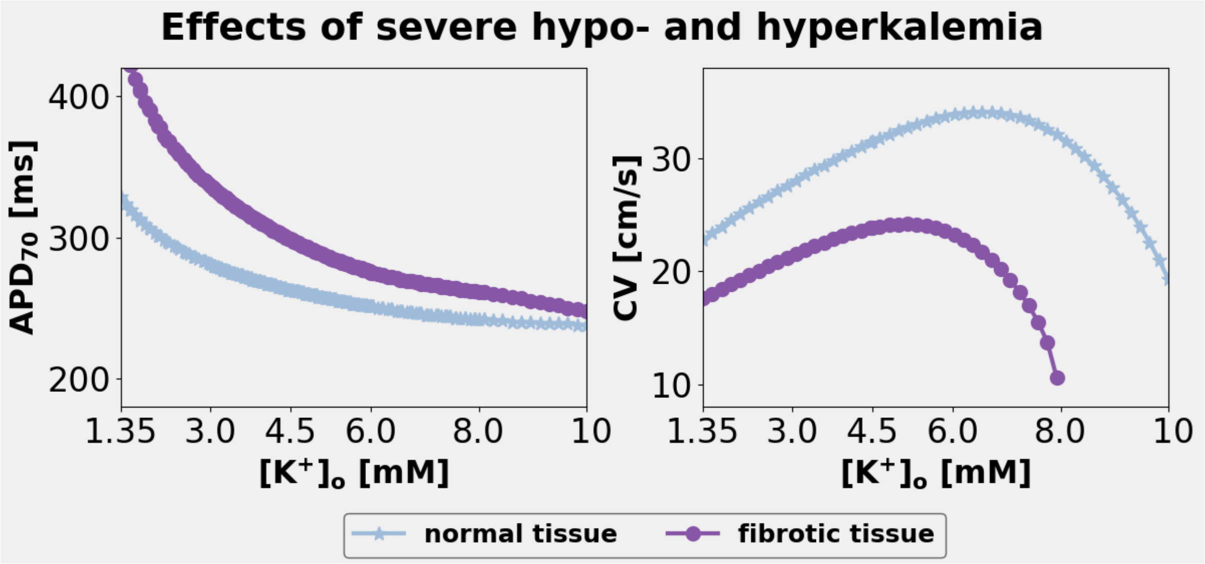

Figure S-7 ***Illustration of the effects of large changes in $[K^+]_o$ on human atrial APD (left) and CV (right) computed under baseline or control conditions (blue) and in the setting of simulated atrial fibrosis (magenta).*** *Variations of $[K^+]_o$ in the 1.25 to 10 mM range were applied one at a time at a cycle length of 1000 ms using the CRN+ model for the human atrial action potential. Note the biphasic effect on CV in the setting of hyperkalemia is accurately reconstructed[12].*

Sensitivity of cardiac safety factor to electrolyte changes

Cardiac safety factor[30] measures the robustness of the conduction, and is expressed as the ratio between the total electrical charge carried by the depolarising current, and the minimum charge required to elicit an action potential. In accordance with the suggestion of one Reviewer, we present its sensitivity to the variations in plasma concentrations adopted in this paper. Focusing on the expression of the true safety factor presented by Boyle et al.[30], we focus our analysis on the variations on the minimum current required to elicit an action potential (I_{thr})

when one concentration at a time is varied. For each alteration in either $[Na^+]_o$,$[K^+]_o$ or $[Ca^{2+}]_o$, we reached a limit cycle by pacing the atrial myocyte at a fixed cycle length and for 15 minutes. Then, we determine the minimum amplitude that elicit an action potential using a bisection method with amplitude in the initial range [0, 250] pA/pF and a stopping tolerance of 1 pA/pF. We computed I_{thr} for a stimulus with durations between 1 ms and 5.5 ms, 0.5 ms apart. We tested a cycle length of 1000 ms representing sinus rhythm and a cycle length of 300 ms, representing an arrhythmia.

As shown in Figure S-8, at a CL of 1000 ms, a smaller stimulus amplitude is required to elicit an action potential when $[K^+]_o$ is increased, while the amplitude required is larger during hypokalaemia. This difference might be due to the smaller `gap ' or voltage difference between the threshold potential and the RMP, since hyponatremia hyperpolarises the RMP. At a CL of 300 ms, we observed an opposite phenomenon. We are not certain of the mechanistic explanations, however, at CL=300 ms the delayed rectifier current exhibits 'memory' or 'residual activation' thus adding a rate-dependent net outward K+ current within the relative refractory period `time window`.

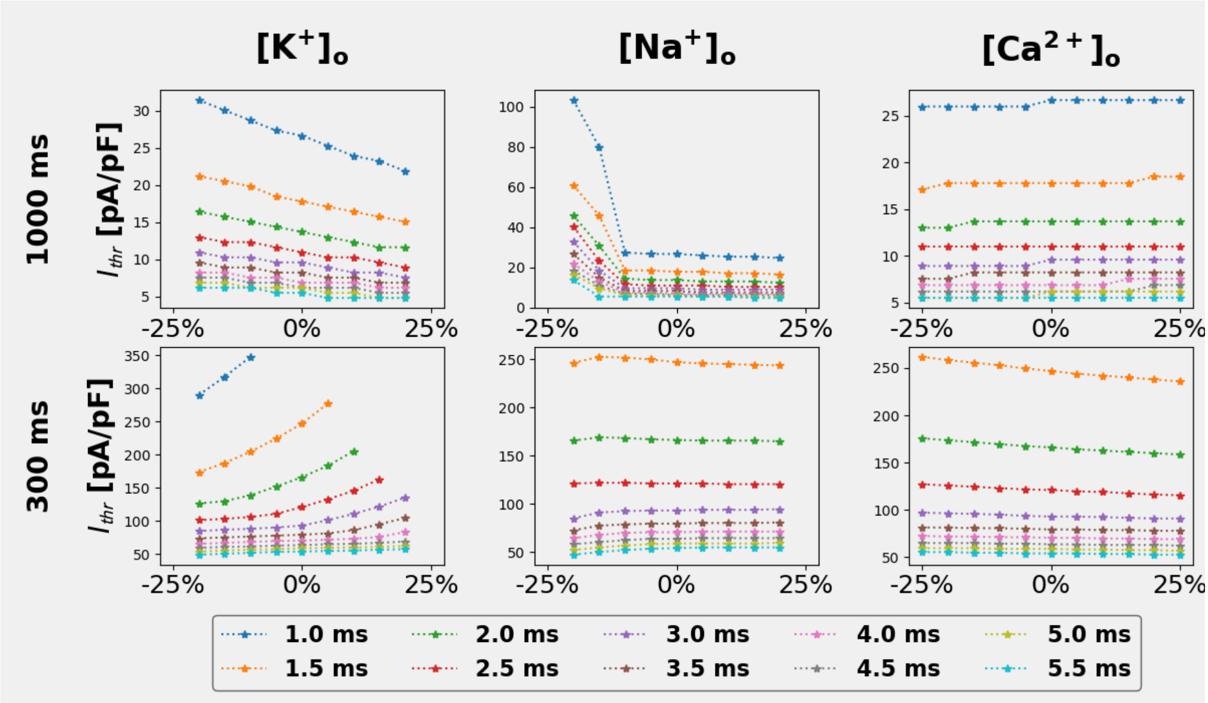

Figure S-8 ***Minimum stimulus amplitude(I_{thr}) required to elicit an action potential****. Colour-coded lines represent a different stimulus duration. A CL of 1000 ms (top row) and 300 ms (bottom row), have been tested for +/-25% variations from the baseline in $[K^+]_o$ (left column), $[Na^+]_o$ (central column), and $[Ca^{2+}]_o$ (right column).*

Summary of atrial simulations and classifier performances

Row #		Induced from LAA	Induced from AR	Induced (any location)	Terminated
A	No. of samples with True as outcome	380 (12.3%)	382 (12.3%)	608 (19.6%)	1083 (35%)
B	No. of trained classifiers	63	61	77	99
C	R^2 score (case by case)	.96±.04 [.96-1.0]	.96±.04 [.96-1.0]	.95±.05 [.95-1.0]	.95±.05 [.95-1.0]
D	R^2 score (overall)	0.82	0.83	0.82	0.87

Table S-3 ***Summary of Atrial Arrhythmia Simulations with Associated Trained Classifiers and r2 Scores*** *These data sets summarise the extensive analyses of each of the effects of changing plasma electrolytes on the initiation and sustainability of model-generated atrial arrhythmias under baseline conditions and in the setting of simulated fibrosis. The data in Row A provide the total number of simulations presenting as True outcomes, including the entire dataset for each binary classification. Note that the chosen burst pacing manoeuvre delivered in the proximity of the LAA successfully induced arrhythmias in 380 out of a total of 3,100 models. The same burst pacing applied to the atrial roof (AR) induced arrhythmias in 382 of the 3,100 models. In 608 of 3,100 models, it was possible to induce arrhythmias using this burst pacing protocol. However, in 1,083 of the 3,100 models, none of the three stimulus protocols induced arrhythmias that lasted at least five seconds, as required for our analysis. Row B in this Table provides information concerning the total number of clinical cases that could be assigned an associated classifier. For each such classifier, training procedures were applied only to those cases that included at least two samples for any of the two outcomes. The data in Row C present* R^2 *scores (mean, standard deviation, and range) of the classifiers trained for data from each human atrial dataset. Finally, the data in Row D show the* R^2 *scores of the classifier trained using the entire clinical dataset after input features were augmented based on the amount of assumed fibrosis (see Table S-1) and the relative surface area.*

Case-by-case sensitivity of atrial arrhythmia properties to variations in plasma concentrations

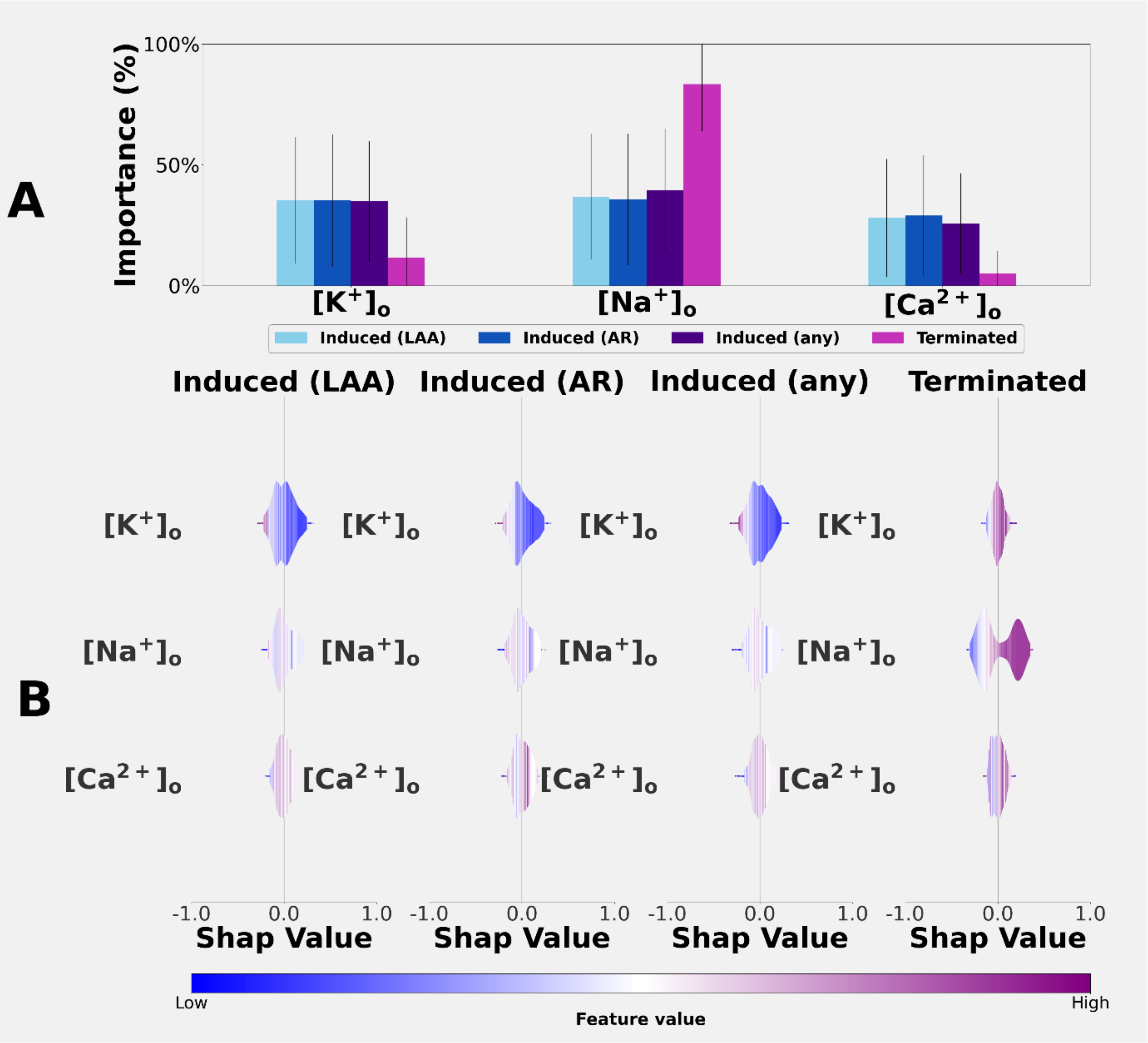

Figure S-9 ***Histograms showing the feature importance (Panel A) and violin plots(Panel B) based on Shapley indices, computed case-by-case and summarised, of selected changes in [K$^+$]$_o$, [Na$^+$]$_o$, and [Ca^{2+}]$_o$ concentrations.*** *We considered the following endpoints: arrhythmia induced by a burst pacing in the proximity of the left atrial appendage (LAA), arrhythmia induced by a burst pacing in the proximity of the left atrial roof (AR), arrhythmia induced by a burst pacing, either on LAA or on AR (any), and arrhythmia terminated within 5 seconds from initiation. Note that induced arrhythmias termination strongly depended on [Na$^+$]$_o$, while no single effect was determinant for the initiation. Hypokalaemia produced a pro-arrhythmic substrate, although this alteration alone was not sufficient to induce arrhythmias.*

Supplementary Electrophysiology background

Illustrations of the effects of changes in plasma electrolytes on the ion transfer (I-V) curves for IK1 and INaCa

Our analysis has emphasised important functional roles for two transmembrane ionic currents, IK1 and INaCa, when attempting to identify principal mechanisms through which plasma electrolyte changes can alter human atrial excitability, action potential waveforms and

susceptibility to the induction and/or maintenance (expressed as `termination`) of human atrial rhythm disturbances. In Figure S-10, the primary drivers for these responses are shown in the form of the respective current-voltage relations (I-V curves) for IK1 and INaCa. In the first part of this analysis, we modified IK1 only, while leaving the mathematical descriptors for INaCa exchanger as published in the original Courtemanche model[1]. These equations are shown below.

$$IK1 = \sqrt{\frac{[K^+]_o}{5.4}} \frac{V - E_k}{1 + exp\big(0.07(V + 80)\big)}$$

$$INaCa = \frac{I_{NaCa(\max)}\left([Na^+]_i^3[Ca^{2+}]_o \exp\left(\frac{\gamma FV}{RT}\right) - [Na^+]_o^3[Ca^{2+}]_i \exp\left(\frac{(\gamma - 1)FV}{RT}\right)\right)}{\left(\mathrm{K}_{m,Na}^3 + [Na^+]_o^3\right)\left(K_{m,Ca} + [Ca^{2+}]_o\right)\left(1 + k_{sat} \exp\left(\frac{(\gamma - 1)FV}{RT}\right)\right)}$$

Families of I-V curves generated in response to ±25% changes in the 3 selected plasma electrolytes are shown in Figure S-10. The left column shows the changes in IK1 in response to changes in $[K^+]_o$. Data in the middle column show changes in INaCa in response to selected alterations in $[Na^+]_o$. The data sets in the right-hand column illustrate changes in INaCa produced by the designated changes in $[Ca^{2+}]_o$.

Note that even small changes in $[K^+]_o$ (~1 mM) can significantly (~40%) alter the outward current generated by IK1. This, in turn, can cause substantial shifts in the RMP. In atrial myocytes and tissue even minor changes in RMP (on the order of 3-5 mV) can produce significant (~30%) changes in INa because the foot of the steady-state inactivation curve for this current is at the same potential as the RMP. Accordingly, small changes in $[K^+]_o$ also have a strong influence on CV.

As shown in the middle and right Panels of Figure S-10 changes in $[Na^+]_o$ quite strongly affect INaCa. by shifting its reversal potential and also by increasing the amount of outward current that can be generated by this transport mechanism in the physiological range of potentials for example at 0 mV. This at least partially explains the strong influence of changes in $[Na^+]_o$ on the action potential waveform and also on APD. In contrast, effects on INaCa produced by changes in $[Ca^{2+}]_o$ appear to be quite modest.

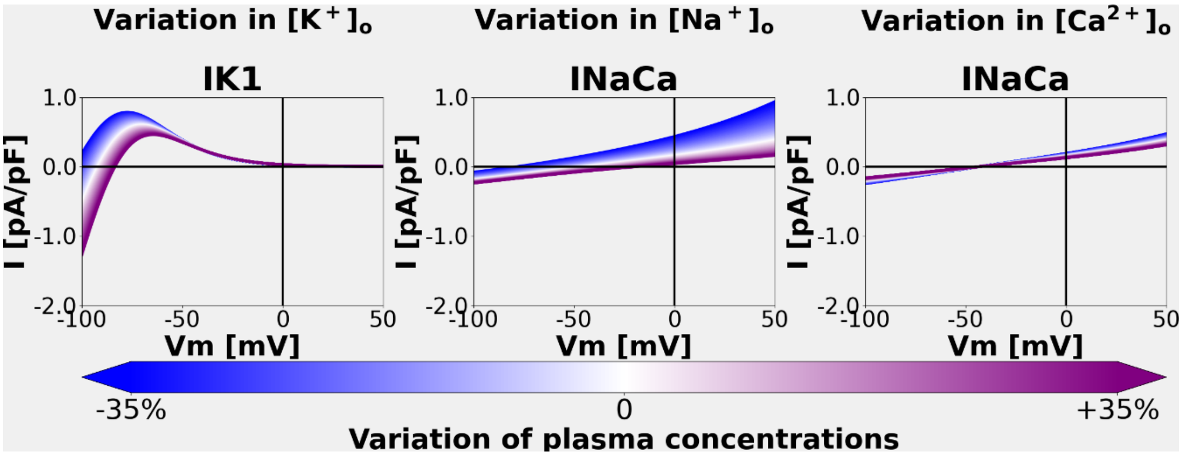

Figure S-10 ***Superimposed current-voltage (I-V) relationship for IK1 and INaCa generated in response to ± 25% changes in $[K^+]_o$, $[Na^+]_o$ or $[Ca^{2+}]_o$ as illustrated at the bottom of this Figure.*** *Each individual I-V curve was generated using 1 selected concentration of either $[K^+]_o$ (left), $[Na^+]_o$ (middle) or $[Ca^{2+}]_o$ (right).*

Calcium dynamics

"The CRN++ model still captures Ca^{2+}-induced Ca^{2+} release in only a simplified manner. Indeed, this model represents the calcium induced calcium release (CICR) in a more phenomenological than mechanistic in the CRN model; and, moreover, it uses a fixed value for the reversal potential (E_{Ca}) in the expression that characterises the L-type Ca^{2+} current (ICaL).

In accordance with the suggestion of one Reviewer, we repeated the analysis presented for a single myocyte: i) using the model introduced by Colman et al.[31] (Col), that adopts a more mechanistic description of CICR, while sharing most of the ion current formulations with the CRN++ model (Figure S-11), and ii) adopting a Nernst potential to characterise E_{Ca} in both models:

$$E_{Ca} = \frac{RT}{2F} \log\left(\frac{[Ca^{2+}]_o}{[Ca^{2+}]_i}\right)$$

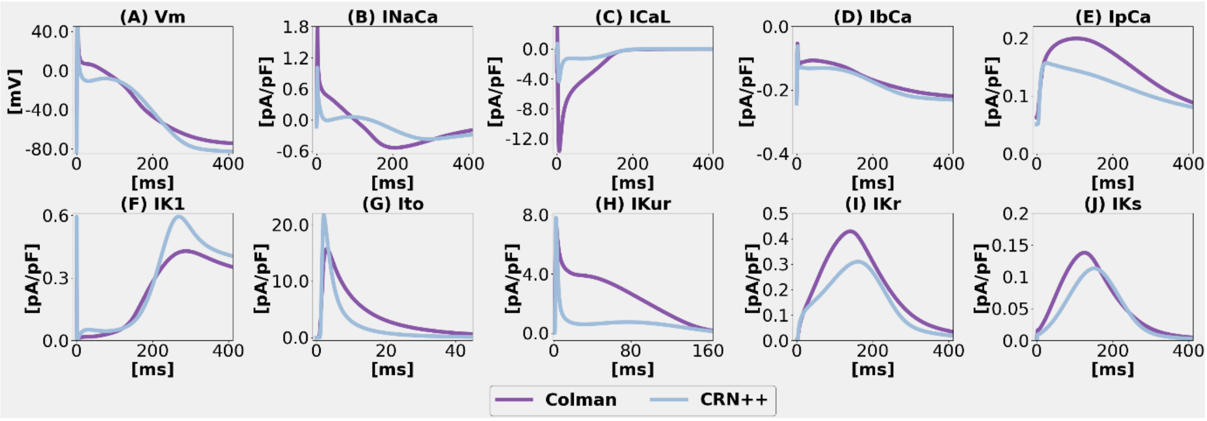

Figure S-11 ***Comparison between Colman (magenta line) and CRN++ (blue line) models for AP, Ca^{2+}-dependent and K^+-dependent currents***

Figure S-12 compares the action potential waveforms generated by CRN++ (Row A) and Col (Row C), when a fixed value of 65 mV is adopted for E_{Ca}; and then, describing E_{Ca} as a Nerst potential (Rows B and D). However, none of these changes produced significant differences in the pattern of results generated by CRN++. The most likely reason is that the small changes in $[Ca^{2+}]_o$ in this study (+/- 30% from the baseline, or approximately 0.5 mM) altered the peak Ca^{2+} current insignificantly.

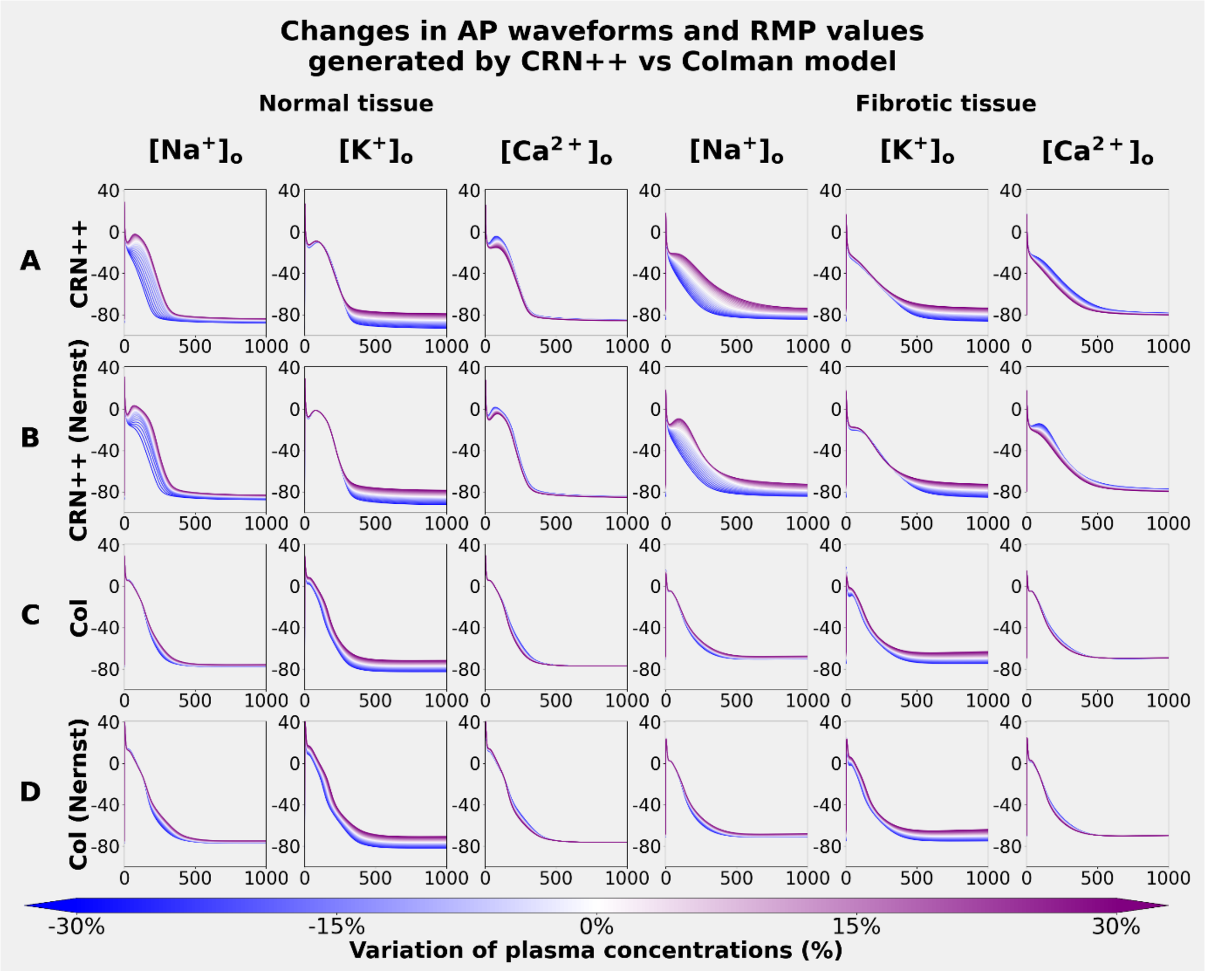

Figure S-12 ***Variations is transmembrane potentials for alterations in $[Na^+]_o$, $[K^+]_o$, and $[Ca^{2+}]_o$ for normal tissue (first 3 columns) and in the settings of fibrosis (last 3 columns) .*** *Row A: CRN++ model. Row B: CRN++ model with the reversal potential for ICaL formulated as a variable $[Ca^{2+}]_i$-dependent parameter (Nernst potential). Row C: Colman (Col) model. Row D: Col model with the reversal potential in ICaL expressed as a variable $[Ca^{2+}]_i$-dependent parameter (Nernst potential).*

As illustrated in Figure S-11, Col (magenta line) exhibits an L-type calcium current nearly twice as large as that in the CRN++ model, with notably slow inactivation (Panel C). This feature causes the model to display a pronounced dependence of the action potential plateau waveform on the L-type calcium current, particularly its slow inactivation dynamics.

References

1. Courtemanche, M., Ramirez, R. J. & Nattel, S. Ionic mechanisms underlying human atrial action potential properties: insights from a mathematical model. *American Journal of Physiology-Heart and Circulatory Physiology* **275**, H301–H321 (1998).

2. Roney, C. H. *et al.* In silico Comparison of Left Atrial Ablation Techniques That Target the Anatomical, Structural, and Electrical Substrates of Atrial Fibrillation. *Front Physiol* **11**, (2020).

3. Corrado, C. *et al.* Quantifying the impact of shape uncertainty on predicted arrhythmias. *Comput Biol Med* **153**, 106528 (2023).

4. Zahid, S. *et al.* Patient-derived models link re-entrant driver localization in atrial fibrillation to fibrosis spatial pattern. *Cardiovasc Res* **110**, 443–454 (2016).

5. Roney, C. H. *et al.* Predicting atrial fibrillation recurrence by combining population data and virtual cohorts of patient-specific left atrial models. *Circ Arrhythm Electrophysiol* **15**, e010253 (2022).

6. Roney, C. H. *et al.* Modelling methodology of atrial fibrosis affects rotor dynamics and electrograms. *EP Europace* **18**, iv146–iv155 (2016).

7. Hakim, J. B., Murphy, M. J., Trayanova, N. A. & Boyle, P. M. Arrhythmia dynamics in computational models of the atria following virtual ablation of re-entrant drivers. *EP Europace* **20**, iii45–iii54 (2018).

8. Deng, D. *et al.* Sensitivity of reentrant driver localization to electrophysiological parameter variability in image-based computational models of persistent atrial fibrillation sustained by a fibrotic substrate. *Chaos: An Interdisciplinary Journal of Nonlinear Science* **27**, 093932 (2017).

9. Boyle, P. M. *et al.* Computationally guided personalized targeted ablation of persistent atrial fibrillation. *Nat Biomed Eng* **3**, 870–879 (2019).

10. Clerx, M., Mirams, G. R., Rogers, A. J., Narayan, S. M. & Giles, W. R. Immediate and delayed response of simulated human atrial myocytes to clinically-relevant hypokalemia. *Front Physiol* **12**, 651162 (2021).

11. Kraft, L. F., Katholi, R. E., Woods, W. T. & James, T. N. Attenuation by magnesium of the electrophysiologic effects of hyperkalemia on human and canine heart cells. *Am J Cardiol* **45**, 1189–1195 (1980).

12. Weiss, J. N., Qu, Z. & Shivkumar, K. Electrophysiology of hypokalemia and hyperkalemia. *Circ Arrhythm Electrophysiol* **10**, e004667 (2017).

13. Tse, G. *et al.* Arrhythmogenic Mechanisms in Hypokalaemia: Insights From Pre-clinical Models. *Front Cardiovasc Med* **8**, (2021).

14. Trenor, B. *et al.* Pro-arrhythmic effects of low plasma [K+] in human ventricle: An illustrated review. *Trends Cardiovasc Med* **28**, 233–242 (2018).

15. Grandi, E., Pasqualini, F. S. & Bers, D. M. A novel computational model of the human ventricular action potential and Ca transient. *J Mol Cell Cardiol* **48**, 112–121 (2010).

16. Grandi, E. *et al.* Human atrial action potential and Ca 2+ model: Sinus rhythm and chronic atrial fibrillation. *Circ Res* **109**, 1055–1066 (2011).

17. Voigt, N. *et al.* Impaired Na+-dependent regulation of acetylcholine-activated inward-rectifier K+ current modulates action potential rate dependence in patients with chronic atrial fibrillation. *J Mol Cell Cardiol* **61**, 142–152 (2013).

18. Thier, S. O. Potassium physiology. *Am J Med* **80**, 3–7 (1986).

19. Collins, A. J. *et al.* Association of serum potassium with all-cause mortality in patients with and without heart failure, chronic kidney disease, and/or diabetes. *Am J Nephrol* **46**, 213–221 (2017).

20. Bouchard, R., Clark, R. B., Juhasz, A. E. & Giles, W. R. Changes in extracellular K+ concentration modulate contractility of rat and rabbit cardiac myocytes via the inward rectifier K+ current IK1. *Journal of Physiology* **556**, 773–790 (2004).

21. Saltelli, A. *et al.* Variance based sensitivity analysis of model output. Design and estimator for the total sensitivity index. *Comput Phys Commun* **181**, 259–270 (2010).

22. Herman, J. & Usher, W. SALib: An open-source Python library for Sensitivity Analysis. *The Journal of Open Source Software* **2**, 97 (2017).

23. Pedregosa, F. *et al.* Scikit-learn: Machine learning in Python. *Journal of Machine Learning Research* **12**, 2825–2830 (2011).

24. Tomek, J. *et al.* Development, calibration, and validation of a novel human ventricular myocyte model in health, disease, and drug block. *Elife* **8**, e48890 (2019).

25. Cordeiro, J. M. *et al.* Regional variation of the inwardly rectifying potassium current in the canine heart and the contributions to differences in action potential repolarization. *J Mol Cell Cardiol* **84**, 52–60 (2015).

26. Giles, W. R. & Imaizumi, Y. Comparison of potassium currents in rabbit atrial and ventricular cells. *J Physiol* **405**, 123–145 (1988).

27. Horváth, A. *et al.* Low Resting Membrane Potential and Low Inward Rectifier Potassium Currents Are Not Inherent Features of hiPSC-Derived Cardiomyocytes. *Stem Cell Reports* **10**, 822–833 (2018).

28. Buiten, M. S. *et al.* The dialysis procedure as a trigger for atrial fibrillation: New insights in the development of atrial fibrillation in dialysis patients. *Heart* **100**, 685–690 (2014).

29. Miyata, A., Dowell, J. D., Zipes, D. P. & Rubart, M. Rate-dependent [K] o accumulation in canine right atria in vivo: electrophysiological consequences. *Am J Physiol Heart Circ Physiol* **283**, H506–H517 (2002).

30. Boyle, P. M. *et al.* New insights on the cardiac safety factor: Unraveling the relationship between conduction velocity and robustness of propagation. *J Mol Cell Cardiol* **128**, 117–128 (2019).

31. Colman, M. A. *et al.* Pro-arrhythmogenic effects of atrial fibrillation-induced electrical remodelling: Insights from the three-dimensional virtual human atria. *Journal of Physiology* **591**, 4249–4272 (2013).
