# Supplementary material for: The effect of clinically relevant changes in extracellular electrolyte concentrations on human atrial arrhythmias
Source: Commun Med (Lond). 2025 Dec 2;6:7. doi: 10.1038/s43856-025-01260-4 (PMC12770435; doi:10.1038/s43856-025-01260-4)
Supplement: Supplementary file 2 — Description of additional supplementary data [file 43856_2025_1260_MOESM2_ESM.pdf]

Description of Additional Supplementary Files

File Name: Supplementary Data 1

Description: Sobol indices APD sensitivity analysis (Figure 3). Data for each panel of Figure 3 is provided as a separate tab in the data file. The first column in each tab identifies the tissue type.

File Name: Supplementary Data 2

Description: Sobol indices CV sensitivity analysis (Figures 4 and 5). Data for figure 3 are provided in the tab *Ko_Nao_Cao*. The first column identifies the tissue type and the cycle length. Data for the Panels in Figure 4 are provided in tabs *Ko*, *Nao* and *Cao*.

File Name: Supplementary Data 3

Description: Sobol indices for the APD and CV (median and inter-percentile range) sensitivity analysis of the atrial surface during paced activation (Figure 6). The first column identifies the quantity of interest.

File Name: Supplementary Data 4

Description: Data for the bar plot of the feature permutation importance (Figure 7, Panel A). The tab *summary* provides the data to plot the bars (mean value and error). The tabs *Induced (LAA)*, *Induced (LAA)*, *Induced (any)*, and *Terminated* contain the values of each tested feature permutation. We tested n=100 permutations.

File Name: Supplementary Data 5

Description: Data for the violin plot of the Shapley indices (Figure 7, Panel B).Each tab represents one of the endpoints (*Induced (LAA)*, *Induced (LAA)*, *Induced (any)*, and *Terminated*) plotted in figure 7B.
